# Dynamic Sumoylation of a Conserved Transcription Corepressor Prevents Persistent Inclusion Formation during Hyperosmotic Stress

**DOI:** 10.1371/journal.pgen.1005809

**Published:** 2016-01-22

**Authors:** Michelle L. Oeser, Triana Amen, Cory M. Nadel, Amanda I. Bradley, Benjamin J. Reed, Ramon D. Jones, Janani Gopalan, Daniel Kaganovich, Richard G. Gardner

**Affiliations:** 1 Molecular and Cellular Biology Program, University of Washington, Seattle, Washington, United States of America; 2 Department of Pharmacology, University of Washington, Seattle, Washington, United States of America; 3 Alexander Grass Center for Bioengineering, Hebrew University of Jerusalem, Jerusalem, Israel; 4 Department of Cell and Developmental Biology, Alexander Silberman Institute of Life Sciences, Hebrew University of Jerusalem, Jerusalem, Israel; Stanford University, UNITED STATES

## Abstract

Cells are often exposed to physical or chemical stresses that can damage the structures of essential biomolecules. Stress-induced cellular damage can become deleterious if not managed appropriately. Rapid and adaptive responses to stresses are therefore crucial for cell survival. In eukaryotic cells, different stresses trigger post-translational modification of proteins with the small ubiquitin-like modifier SUMO. However, the specific regulatory roles of sumoylation in each stress response are not well understood. Here, we examined the sumoylation events that occur in budding yeast after exposure to hyperosmotic stress. We discovered by proteomic and biochemical analyses that hyperosmotic stress incurs the rapid and transient sumoylation of Cyc8 and Tup1, which together form a conserved transcription corepressor complex that regulates hundreds of genes. Gene expression and cell biological analyses revealed that sumoylation of each protein directs distinct outcomes. In particular, we discovered that Cyc8 sumoylation prevents the persistence of hyperosmotic stress-induced Cyc8-Tup1 inclusions, which involves a glutamine-rich prion domain in Cyc8. We propose that sumoylation protects against persistent inclusion formation during hyperosmotic stress, allowing optimal transcriptional function of the Cyc8-Tup1 complex.

## Author Summary

Cells have evolved complex stress responses to cope with environmental challenges that could otherwise inflict severe damage on the molecules essential for life. Stress responses must ameliorate the immediate damage caused by stress exposure and also adjust metabolic capacity, gene expression output, and other cellular functions to protect against further damage that could be incurred by prolonged exposure to stress. Posttranslational protein modifications are a major means by which cells respond to changing environmental conditions. These modifications can alter the function, localization, and molecular interactions of their target proteins. In addition, evidence is emerging that some posttranslational modifications may also change the physical characteristics of target proteins. In this study, we present evidence that during hyperosmotic stress, a condition known to induce protein misfolding, cells rapidly but transiently use the small ubiquitin-modifier SUMO to protect against persistent inclusion formation of a conserved transcriptional repressor complex. We propose that this rapid protective action via posttranslational modification enables optimal gene regulation during the cellular response to hyperosmotic stress.

## Introduction

Throughout their lives, cells will be exposed to a variety of stresses: extreme temperatures, altered osmolarity, hypoxia, free radicals, infections, and genotoxic insults. Exposure to these stresses can deleteriously damage the structures of essential biomolecules such as DNA, RNA, and proteins. Thus, a cell’s ability to sense, react, and adapt to stress is crucial for survival. Stress responses have evolved to protect the cell in two major ways. Stress responses initiate cellular programs that rapidly alter specific protein activities to cope with the immediate damage caused by acute exposure to stress. They also adjust gene expression and metabolism to protect against further damage that can be incurred by prolonged exposure to stress. Both immediate and sustained stress response mechanisms are essential for directing cellular resources towards repair and protection and away from growth and proliferation. Failure to respond appropriately to stress-induced damage can lead to loss of cell viability. Importantly, many human diseases (e.g. diabetes, heart disease, cancer, and neurodegeneration) result from, or cause cellular stress [1].

Posttranslational protein modifications are a crucial means the cell uses to elicit functional changes during stress. A key protein modification that is an important and immediate signal in response to stress is the small ubiquitin-like modifier SUMO. Similar to ubiquitin, SUMO modification occurs via a multi-enzyme cascade [2,3]. Initially, a SUMO-activating enzyme activates SUMO in an ATP-dependent manner [2]. A SUMO-conjugating enzyme then attaches SUMO to lysine residues of a target protein in collaboration with a SUMO ligase [2]. Sumoylation is reversible and its removal is mediated by desumoylating enzymes [2]. To function optimally as a stress regulator, the addition and removal of SUMO must be dynamically controlled by the cell.

Proteomic studies in many eukaryotes have revealed that dramatic increases in protein sumoylation occur following heat, oxidative, salt, and ethanol stresses [4,5,6,7,8,9,10,11,12,13]. While the identities of proteins subject to stress-induced sumoylation have been catalogued in a number of organisms, in the majority of cases it is not clear what function stress-induced sumoylation serves at the individual protein level. Under normal conditions, sumoylation typically directs changes in protein function, localization, and/or interactions [3,14]. More recently, sumoylation has been found to play a role in protein folding and quality control [4,12,15,16,17]. It remains an open question whether stress-induced sumoylation coordinates canonical regulatory responses or protects the folding state of its protein targets. These outcomes are not mutually exclusive and their involvement will depend upon the specific proteins sumoylated during stress. Here, we uncovered a new role for sumoylation in preventing the highly conserved transcriptional corepressor Cyc8 from forming persistent inclusions during hyperosmotic stress in the budding yeast *Saccharomyces cerevisiae*. We use the term "inclusion" in its broadest definition, which is to describe cellular foci not bounded by membranes that can range from misfolded protein aggregates to concentrated functional protein assemblies.

## Results

### Rapid, dynamic sumoylation changes occur during hyperosmotic stress

**Fig 1:**
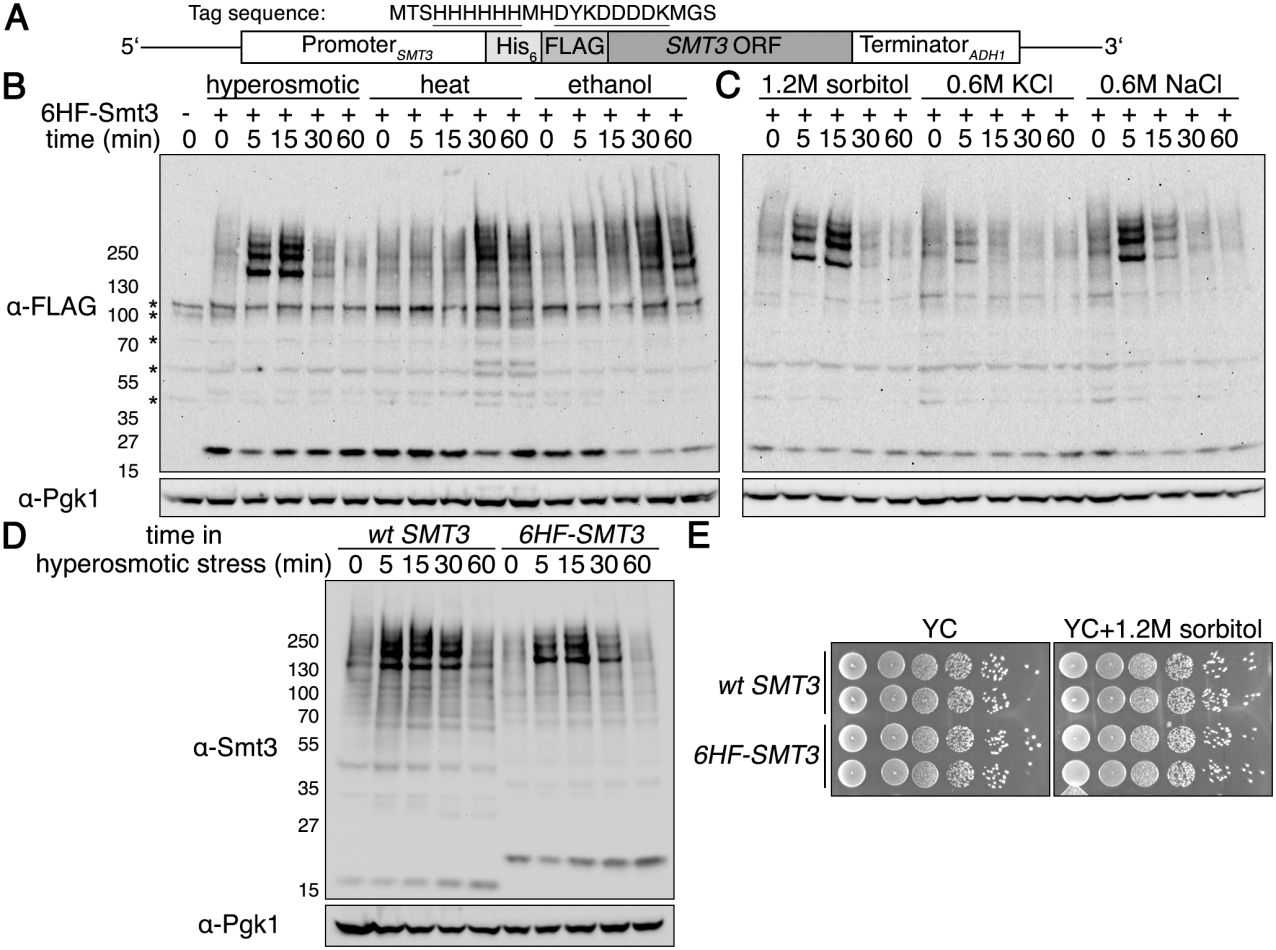
Rapid, transient sumoylation occurs during hyperosmotic stress. (**A**) Schematic for *His*_*6*_*-FLAG-SMT3* located at the genomic *SMT3* locus and expressed from the endogenous *SMT3* promoter. Sequence of the His_6_-FLAG sequence is shown, with the His_6_ and FLAG sequences underlined. The His_6_-FLAG tag is identical to that used previously [22]. (**B**) Comparison of global sumoylation changes that occur during a time course of hyperosmotic stress (1.2M sorbitol), heat shock (42°C), or ethanol stress (10% v/v). Cells expressing His_6_-FLAG-Smt3 (HF-Smt3) were subjected to each stress over a time course of 60 minutes. Changes in sumoylation patterns were examined by western analysis using an anti-FLAG antibody. Cells expressing endogenous Smt3 were used as a control for nonspecific bands, indicated by asterisks on the left. Pgk1 was detected as a loading control. (**C**) Examination of global sumoylation changes during different hyperosmotic stresses. Western analysis was performed as in (B). (**D**) Comparison of global sumoylation changes in wild-type *SMT3* and *His*_*6*_*-FLAG-SMT3* cells during a time course of hyperosmotic stress (1.2M sorbitol). Cell lysates were prepared and proteins separated by SDS-PAGE. Changes in sumoylation patterns were analyzed by western analysis using an anti-Smt3 antibody. (**E**) Addition of the *6His-FLAG* sequence to endogenous *SMT3* does not alter growth under non-stressed and hyperosmotic stress conditions. Wild-type *SMT3* and *His*_*6*_*-FLAG-SMT3* cells were 10-fold serially diluted onto YC plates with and without 1.2M sorbitol and incubated at 30°C for 3 days.

Several previous studies that examined global sumoylation in yeast used a tandem 6x histidine (His_6_), FLAG-epitope tagged version of the yeast SUMO protein Smt3 that was either overexpressed from a plasmid [18,19,20], or expressed at normal levels from the endogenous *SMT3* genomic locus [7]. We wished to avoid any spurious issues that could occur due to overexpression, so we opted for an endogenous expression approach to examine the temporal changes in sumoylation that occur during application of different stresses. We constructed a yeast strain wherein we altered the endogenous *SMT3* gene by adding a *His*_*6*_*-FLAG* coding sequence to its 5’ end (Fig 1A). With this strain, we investigated sumoylation patterns over time in response to various stressors: hyperosmotic stress (1.2M sorbitol), heat shock (42°C), and ethanol stress (10% v/v). Heat shock and ethanol stress resulted in steady accumulation of sumoylation, whereas hyperosmotic stress caused a rapid and transient sumoylation pattern (Fig 1B), similar to what was reported recently [21].

We found the transient hyperosmotic stress-induced sumoylation intriguing. To ensure the effect was consistent, we examined other commonly used hyperosmotic stressors using equal osmolyte concentrations– 0.6M NaCl or 0.6M KCl. We observed identical sumoylation patterns as with 1.2M sorbitol albeit with altered kinetics and amplitudes (Fig 1C), indicating that equal osmolyte concentrations are not equivalent osmolyte stresses sensed by the cell. We verified the transient hyperosmotic stress effect was not an artifact of the His_6_-FLAG fusion to Smt3. Using a native SUMO antibody, we found wild-type *SMT3* cells exhibited the same pattern of dynamic sumoylation during 1.2M sorbitol addition as *His*_*6*_*-FLAG-SMT3* cells (Fig 1D). We also compared the growth of *His*_*6*_*-FLAG-SMT3* cells to wild-type *SMT3* cells in normal or hyperosmotic stress conditions and found no effect of the tag on cell growth (Fig 1E).

### Tup1 and Cyc8 are major proteins sumoylated during hyperosmotic stress

**Fig 2:**
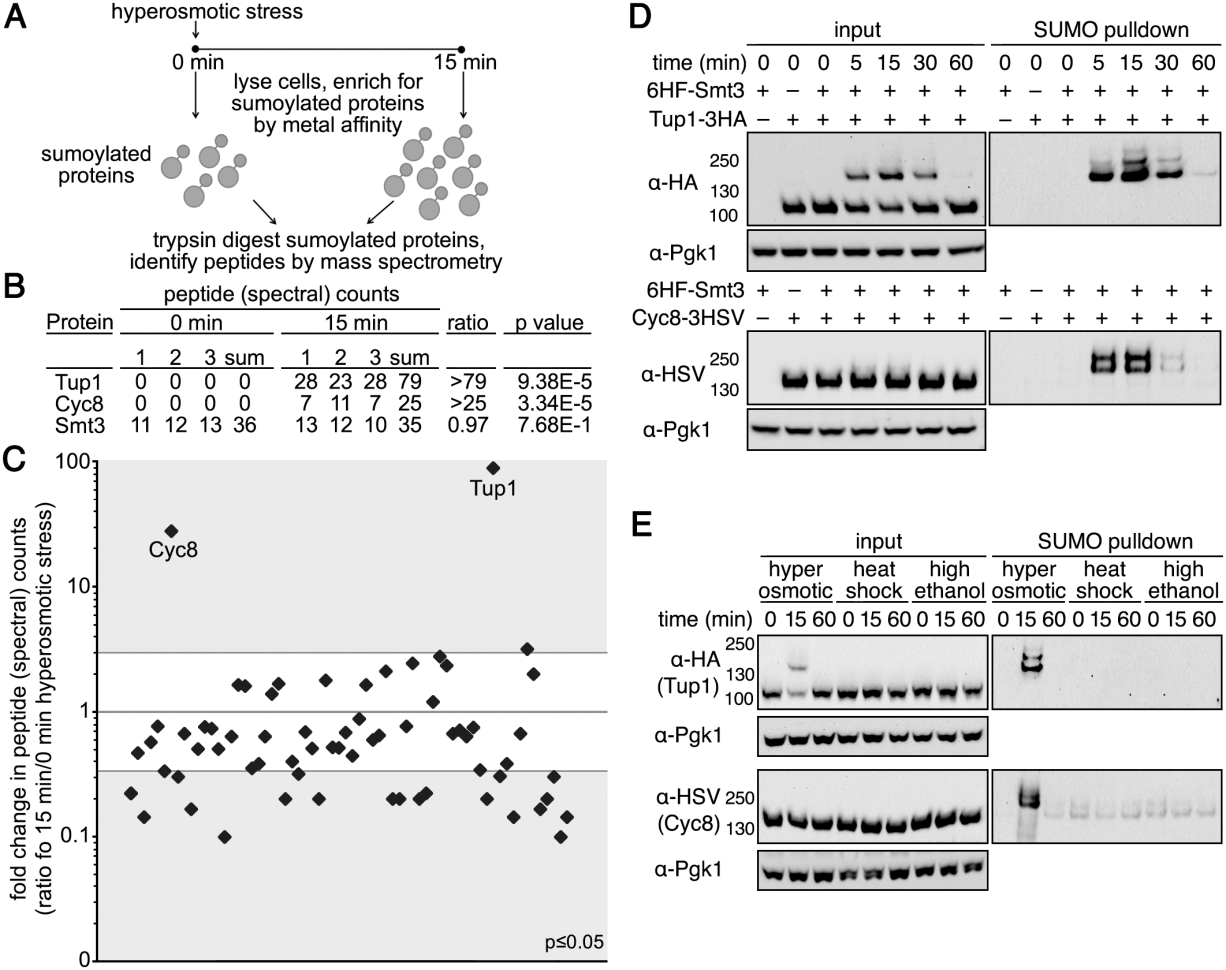
Tup1 and Cyc8 are sumoylated during hyperosmotic stress. (**A**) Scheme of the MS strategy to identify proteins sumoylated during hyperosmotic stress. (**B**) Total peptide counts identified for Tup1 and Cyc8 at 0 and 15 minutes of hyperosmotic stress. Total peptide counts for Smt3 are included to demonstrate equivalent levels of SUMO in the samples. (**C**) Total peptide counts identified for proteins where the significance of the changes between 0 and 15 minutes of hyperosmotic stress was *p*≤0.05. Gray areas represent ≥3 fold changes in the 15 minute samples compared with the 0 minute samples. Tup1 and Cyc8 are noted. (**D**) Cells expressing His_6_-FLAG-Smt3 (HF-Smt3) and either a 3xHA epitope-tagged Tup1 (Tup1-3HA) or a 3xHSV epitope-tagged Cyc8 (Cyc8-3HSV) from their endogenous promoters were subject to hyperosmotic stress (1.2M sorbitol) over a 60-minute time course. Cell lysates (input) and purified sumoylated proteins (SUMO pulldown) were subject to western analyses using anti-HA, anti-HSV, or anti-Pgk1 antibodies to detect Tup1, Cyc8, or Pgk1 respectively. (**E**) Similar experiment as in (D) except cells were subject to hyperosmotic stress (1.2M sorbitol), heat shock (42°C), or high ethanol (10% v/v) for 0, 15, and 60 minutes.

To identify proteins that are sumoylated during hyperosmotic stress, we adopted a label-free mass spectrometry (MS) approach, wherein changes in total peptide (spectral) counts for a protein are a semi-quantitative proxy for protein abundance [23]. Using metal affinity chromatography, we enriched for sumoylated proteins from *His*_*6*_*-FLAG-SMT3* cell lysates generated under heavy denaturing conditions (8M urea and 0.05% SDS) from cultures obtained before and after 15 minutes of hyperosmotic stress (Fig 2A). We specifically chose heavy denaturing conditions so that we could minimize co-purifying proteins that would confound the analysis.

After the MS analysis of peptides generated by trypsin digestion, we categorized a protein as undergoing hyperosmotic stress-dependent sumoylation if the summed peptide counts from the 15-minute replicates exceeded those from the 0-minute replicates by ≥3 fold, and if the peptide counts in the 15-minute replicates were statistically distinct from the 0-minute replicates (*p*≤0.01), similar to our previous studies [24,25]. Upon analyzing the purified sumoylated proteins by MS, we found that two proteins—Tup1 and Cyc8—were strikingly enriched in the 15 minute samples (Fig 2B and 2C and S1 Table). This result was interesting because Tup1 and Cyc8 (aka Ssn6) form a transcription corepressor complex that regulates the transcription of genes involved in carbon metabolism, mating-type switching, and responses to stress [26]. We note that other proteins could be sumoylated during hyperosmotic stress and these could have been missed due to the stringent protein isolation conditions we used for our analysis. Given the dramatic increases in sumoylation with Tup1 and Cyc8 during hyperosmotic stress (Fig 2B and 2C and S1 Table), however, we chose to specifically pursue the function for the sumoylation of these two proteins.

We confirmed that Tup1 and Cyc8 were sumoylated during hyperosmotic stress by enriching for sumoylated proteins from lysates of *His*_*6*_*-FLAG-SMT3* cells and probing specifically for epitope-tagged versions of Tup1 and Cyc8 expressed from their endogenous promoters. Both proteins displayed multiple, transient sumoylated species after hyperosmotic stress (Fig 2D, right panels). Neither protein was sumoylated during heat shock or ethanol stress (Fig 2E, right panels), indicating that the striking increase in sumoylation of Tup1 and Cyc8 is specific for hyperosmotic stress, and not a general SUMO stress response as has been recently proposed [13].

**Fig 3:**
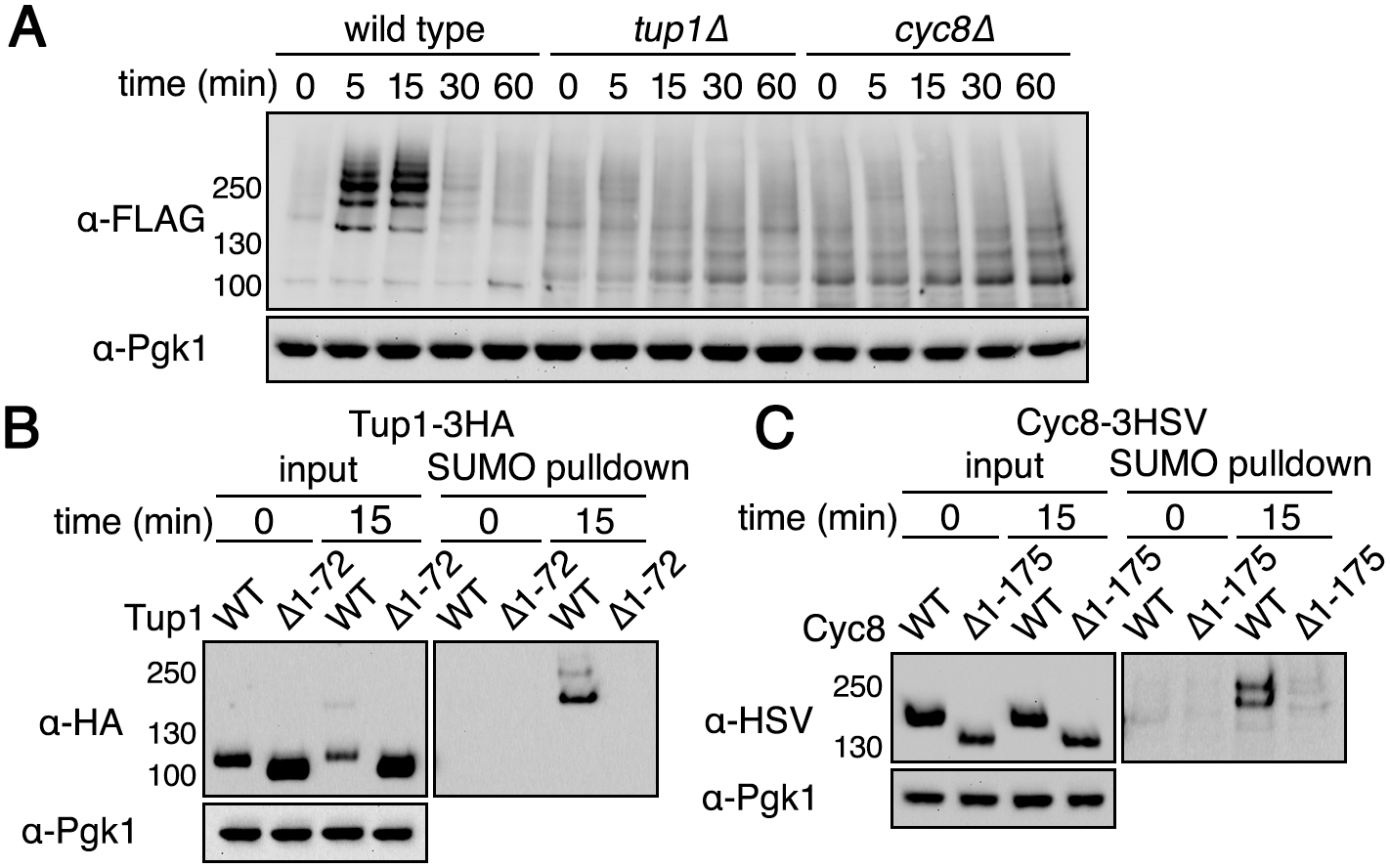
Tup1 and Cyc8 need to be in a complex together for hyperosmotic stress-dependent sumoylation. (**A**) *His*_*6*_*-FLAG-SMT3* wild-type, *tup1*Δ, and *cyc8* cells were subjected to hyperosmotic stress (1.2M sorbitol) over a time course of 60 minutes. Changes in sumoylation patterns were examined by western analysis using an anti-FLAG antibody. (**B-C**) *His*_*6*_*-FLAG-SMT3* promoters were subject to hyperosmotic stress (1.2M sorbitol) for 15 minutes. Cell lysates were generated and subject to metal affinity chromatography to purify sumoylated proteins. Cell lysates (input) and purified sumoylated proteins (SUMO pulldown) were subject to SDS-PAGE and western analyses using anti-HA (Tup1) or anti-HSV (Cyc8) antibodies. (**B**) Results for Tup1-3HA sumoylation when the Cyc8-interaction region was deleted in Tup1. (**C**) Results for Cyc8-3HSV sumoylation when the Tup1-interaction region was deleted in Cyc8.

We deleted each gene to determine the extent to which sumoylated Tup1 and Cyc8 contributed to the overall amount of sumoylated species observed during hyperosmotic stress. Surprisingly, deletion of either *TUP1* or *CYC8* abolished the dramatic sumoylation increase normally observed during hyperosmotic stress (Fig 3A). This is consistent with the MS data showing that Tup1 and Cyc8 are the major sumoylated proteins (Fig 2C). We note that there was a higher background of sumoylation in the absence of either Tup1 or Cyc8. It is possible that without Tup1 or Cyc8 expression, there is more SUMO that is free to be added to other proteins. It is also possible that gene derepression in the absence of Cyc8-Tup1 function leads to more observable background sumoylation of the new proteins produced. These possibilities are not mutually exclusive.

The vastly reduced sumoylation response to hyperosmotic stress in *tup1*Δ and *cyc8*Δ cells suggested that Tup1 and Cyc8 might need to be in a complex for their individual sumoylation, such that deletion of one abrogates the sumoylation of the other. We addressed this possibility by using mutant forms of Tup1 and Cyc8 that cannot interact with each other. In Tup1, residues 1–72 mediate the interaction with Cyc8 [27]. In Cyc8, residues 1–175 mediate the interaction with Tup1 [28]. We expressed epitope-tagged versions of either wild-type Tup1 or Tup1^Δ1–72^ and wild-type Cyc8 or Cyc8^Δ1–175^ in a strain in which endogenous untagged Tup1 and Cyc8 were both present. This configuration allowed for Tup1-Cyc8 complexes to form normally in the cell and avoided any phenotypic complications that might result from complete loss of the complex function that occurs with the full deletion strains. Using this strategy, we found that only wild-type Tup1 and Cyc8, but not mutant Tup^Δ1–72^ or Cyc8^Δ1–175^, were sumoylated during hyperosmotic stress (Fig 3B and 3C). These results support the idea that Tup1 and Cyc8 need to be in a complex together for their sumoylation.

**Fig 4:**
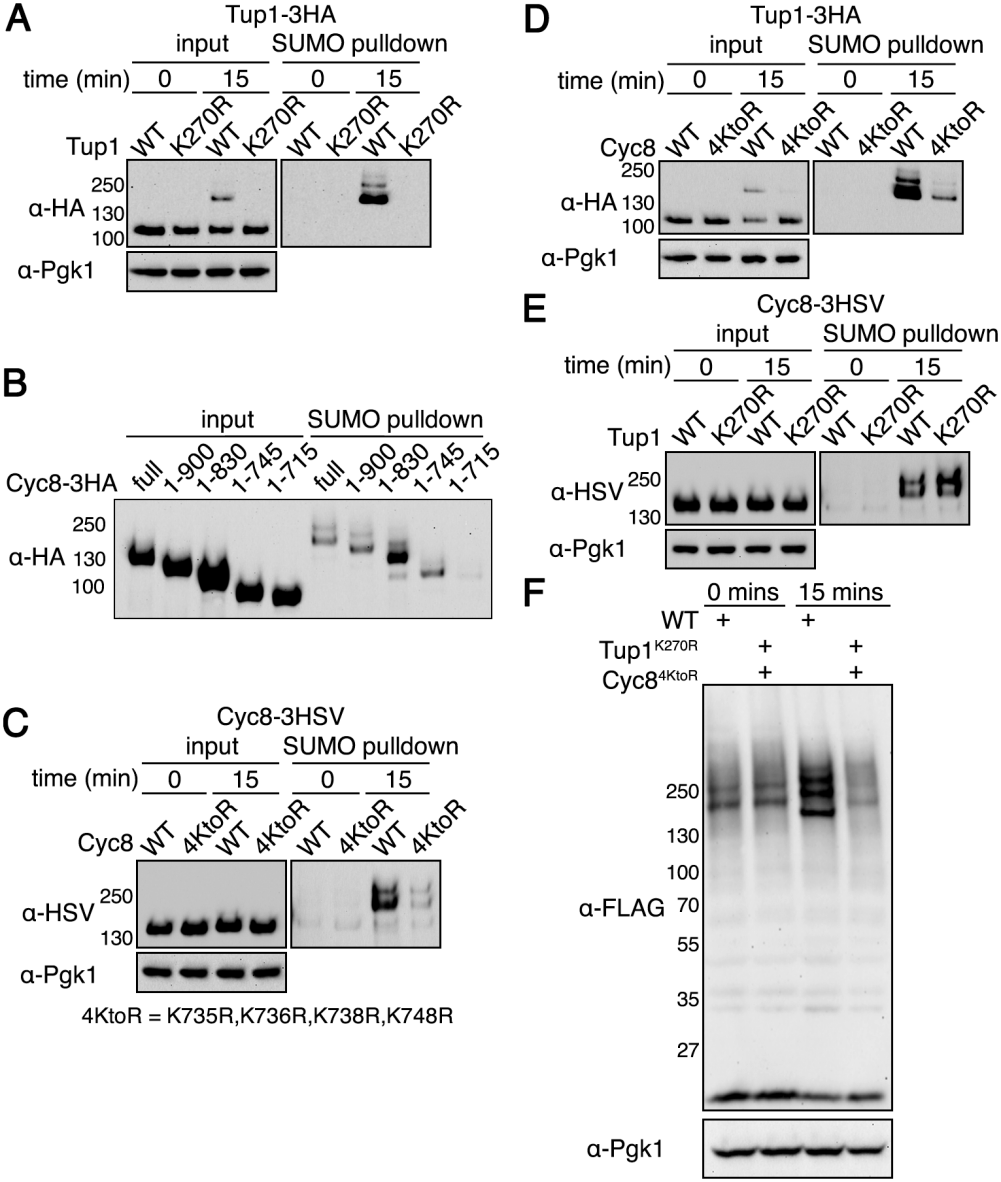
Identification of Tup1 and Cyc8 sumoylation sites. (**A**) *His*_*6*_*-FLAG-SMT3* cells expressing either wild-type Tup1-3HA or Tup1^K270R^-3HA from the endogenous *TUP1* promoter were examined for Tup1 sumoylation at 0 or 15 minutes of hyperosmotic stress (1.2M sorbitol) as in (Fig 2D). (**B**) *His*_*6*_*-FLAG-SMT3* cells expressing the indicated Cyc8-3HSV deletion mutant were subject to hyperosmotic stress (1.2M sorbitol) for 15 minutes. Cell lysates were generated and subject to the same analysis as in (Fig 2D). (**C**) *His*_*6*_*-FLAG-SMT3* cells expressing either wild-type Cyc8-3HSV or Cyc8^4KtoR^-3HSV from the endogenous *CYC8* promoter were examined for Cyc8 sumoylation as in (Fig 2D). (**D**) *His*_*6*_*-FLAG-SMT3* cells expressing wild-type Tup1-3HA from the endogenous *TUP1* promoter and either wild-type Cyc8-3HSV or Cyc8^4KtoR^-3HSV from the endogenous *CYC8* promoter were examined for Tup1 sumoylation as in (A). (**E**) *His*_*6*_*-FLAG-SMT3* cells expressing wild-type Cyc8-3HSV from the endogenous *CYC8* promoter and either wild-type Tup1-3HA or Tup1^K270R^-3HA from the endogenous *TUP1* promoter were examined for Cyc8 sumoylation as in (C). (**F**) *6His-FLAG-SMT3* wild-type or *tup1*^*K270R*^*cyc8*^*4KtoR*^ cells were subjected to hyperosmotic stress (1.2M sorbitol) for 0 or 15 minutes, and global sumoylation patterns examined by western analysis using an anti-FLAG antibody.

It was possible that Tup1^Δ1–72^ and Cyc8^Δ1–175^ were not sumoylated because the deleted regions contain the sites of sumoylation. Therefore, we proceeded to identify the sites of Tup1 and Cyc8 sumoylation during hyperosmotic stress. It was previously reported that Tup1 is sumoylated at low levels on lysine 270 in normal growth conditions [29]. We generated an epitope-tagged Tup1 with the corresponding K270R mutation (Tup1^K270R^). Mutation of lysine 270 completely abolished Tup1 sumoylation during hyperosmotic stress (Fig 4A), indicating that lysine 270 is the sole site modified by SUMO in response to hyperosmotic stress. For Cyc8, low levels of sumoylation have been demonstrated during normal growth conditions [30,31], but the precise sites of Cyc8 sumoylation are unknown. Cyc8 contains 32 lysines, and we found that 3 lysine residues C-terminal to position 735 were predicted to be strong SUMO consensus sites using a SUMO site prediction algorithm [32]. However, individual mutation of these residues did not alter Cyc8 sumoylation. To determine the location of Cyc8’s sumoylation sites, we made C-terminal truncation mutants of Cyc8. We found Cyc8 sumoylation was greatly reduced when the protein was truncated from residues 745 to 715 (Fig 4B). We mutated the lysine residues in this region (lysines 735, 736, and 738) and a proximal lysine residue at 748 to arginine (now called Cyc8^4KtoR^). After doing so, we observed a dramatic reduction in the hyperosmotic stress-induced sumoylation of Cyc8 (Fig 4C). Thus, we show that the Tup1 region 1–72 and the Cyc8 region 1–175 do not contain the sites of sumoylation.

Because Tup1 and Cyc8 sumoylation was contingent upon the formation of a Tup1-Cyc8 complex (Fig 3B and 3C), we examined whether Tup1 sumoylation was dependent on Cyc8 sumoylation, or vice versa. Loss of Cyc8 sumoylation considerably reduced the levels of Tup1 sumoylation (Fig 4D). By contrast, loss of Tup1 sumoylation did not alter Cyc8 sumoylation (Fig 4E). We conclude from these data that Cyc8 sumoylation is a controlling modification in the complex.

Using the sumoylation-deficient Tup1 and Cyc8 mutants, we determined the extent of total hyperosmotic stress-induced sumoylation due to Tup1 and Cyc8 sumoylation. Mutation of the Tup1 and Cyc8 sumoylation sites completely abrogated the observable global increase in sumoylation during hyperosmotic stress (Fig 4F). Thus, we conclude that the major observable SUMO conjugates formed in wild-type cells during hyperosmotic stress are Tup1 and Cyc8. We note that other minor hyperosmotic-dependent sumoylated species that are below detection by western analysis could still exist.

### Loss of Tup1 or Cyc8 sumoylation elicits distinct transcriptional effects

Because Tup1 and Cyc8 are the major proteins sumoylated during hyperosmotic stress, we wondered if there were phenotypic consequences of losing sumoylation on Tup1 and/or Cyc8. The main question we wanted to resolve was whether the combined sumoylation of Tup1 and Cyc8 serves a single purpose or if the individual sumoylation of Tup1 and Cyc8 each directs distinct outcomes. We began by examining gene expression changes because Tup1 and Cyc8 form a well-conserved transcriptional corepressor complex [26]. Deletion of *TUP1* or *CYC8* has been shown to affect the expression of ~1000 genes by ≥1.5 fold during unstressed conditions [33]. While there was considerable overlap in gene expression changes between *tup1*Δ and *cyc8*Δ cells in that study, there were also significant differences suggesting a potential separation of function for each protein.

We performed transcript microarray analyses using strains with different combinations of wild-type and sumoylation-deficient Tup1 and/or Cyc8 over a time course of 0, 30, and 60 minutes after 1.2M sorbitol addition. To identify significant changes in gene expression, we compared wild-type replicates and found that 3 standard deviations from the mean corresponded to a 1.57-fold change in gene expression. We used this empirically derived value as the significance cutoff in our analyses.

**Fig 5:**
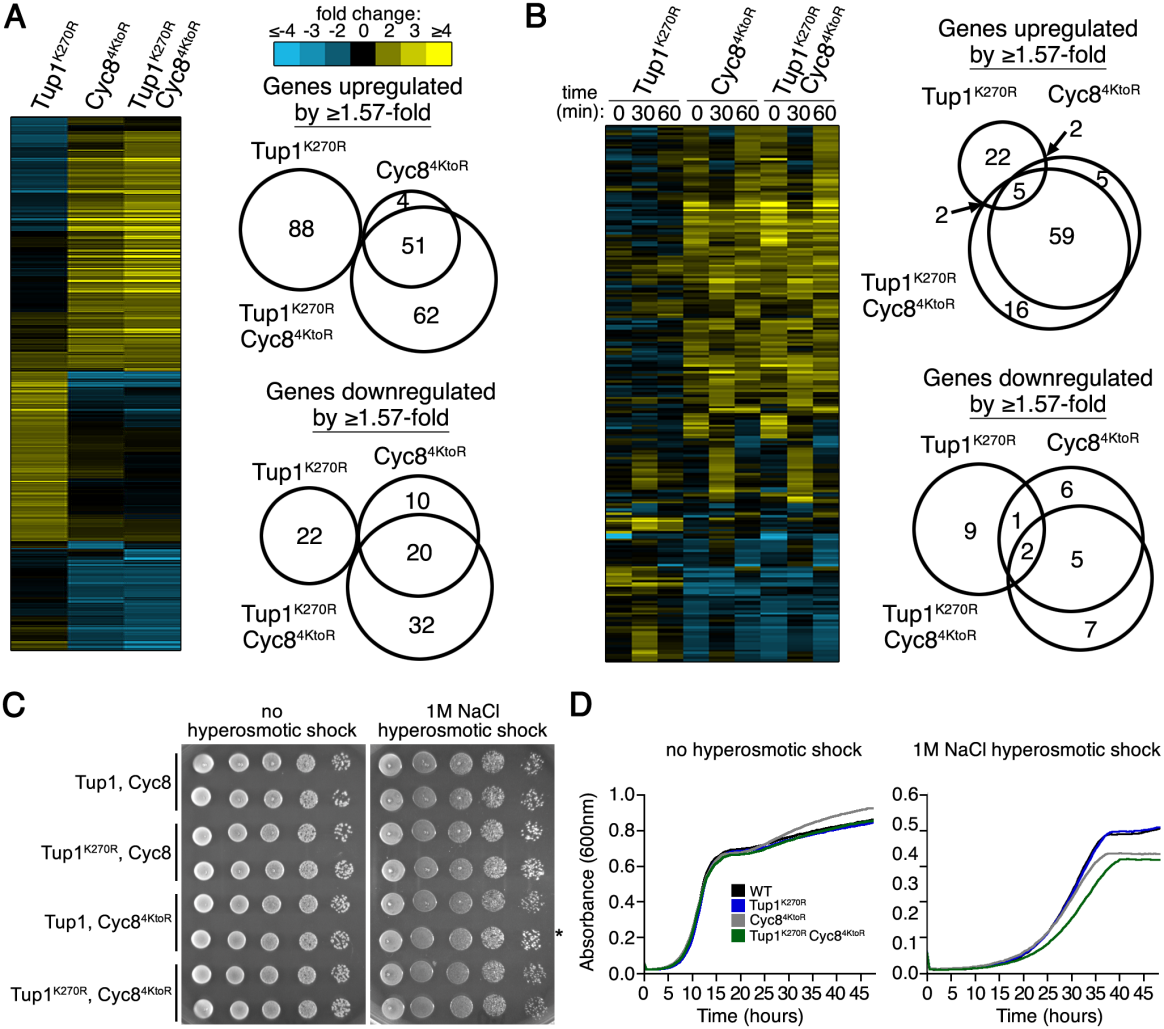
Distinct alterations in transcription occur with the sumoylation-deficient Tup1 and Cyc8 mutants. RNA was collected from biological duplicate samples of wild-type and mutant strains (expressing Tup1^K270R^ and/or Cyc8^4KtoR^ from their endogenous promoters) that were exposed to 0, 30, or 60 minutes of hyperosmotic stress (1.2M sorbitol). Microarray experiments were performed in which the wild-type, unstressed replicate sample served as the reference for comparison with all other samples with a set of replicates. Log_2_-transformed gene expression ratios were averaged across replicate comparisons. (**A**) Hierarchical clustering of gene expression changes that are ≥1.57-fold at the 0 time point. Venn diagrams display overlap between mutant datasets. (**B**) Hierarchical clustering of mutant-effect ratios from gene expression changes that are ≥1.57-fold during 0, 30, and 60 minutes of hyperosmotic stress. Actual gene expressions changes are in S1H Fig. Venn diagrams display overlap between mutant datasets at 30 minutes. (**C**, **D**) Cyc8^4KtoR^ cells have a modest growth deficit in hyperosmotic stress. (**C**) 10-fold dilutions of the indicated strains were spotted on rich media with or without 1M NaCl. Asterisk indicates a Cyc8^4KtoR^ isolate that does not exhibit a growth defect. (**D**) Three independent cultures of the indicated strains were inoculated to an optical density of 0.01 measured by absorbance at 600nm in rich media with or without 1M NaCl. Absorbance values were measured every 5 minutes over the course of 48 hours in a Bioscreen C reader (Growth Curves USA). Curves are the averages of three independent cultures for each strain.

We first examined gene expression changes at the 0 time point because in unstressed conditions Tup1 and Cyc8 regulate many genes [33], and are sumoylated at low levels [29,30]. In our initial analysis, we observed far fewer total changes with the sumoylation-deficient mutants (~290 genes, Fig 5A and S2 Table) than with the gene deletion mutants (~1000, [33]), indicating that loss of sumoylation has much subtler transcriptional effects than the deletions. We confirmed this by examining the ability of Tup1^K270R^ cells and Cyc8^4KtoR^ cells to flocculate, which is a characteristic phenotype of *tup1*Δ cells and *cyc8*Δ cells [26]. Strains deficient in Tup1 or Cyc8 sumoylation did not show a flocculation phenotype (S1A Fig).

The most striking effect we noticed at the 0 time point was that Tup1^K270R^ cells showed a distinct set of gene expression changes compared with Cyc8^4KtoR^ cells (Fig 5A and S1B Fig and S2 Table). In addition, the changes in the double Tup1^K270R^/Cyc8^4KtoR^ strain strongly correlated with the changes observed in the single Cyc8^4KtoR^ strain, indicating that loss of Cyc8 sumoylation is epistatic to loss of Tup1 sumoylation for gene expression. The unstressed expression results provided the first piece of evidence that loss of Tup1 and Cyc8 sumoylation each leads to distinct cellular alterations.

The role of the Tup1-Cyc8 complex in gene repression has been examined for many specific genes under unstressed conditions [34,35,36,37,38,39]. We focused on 16 example genes from these studies that were uniformly derepressed when *TUP1* or *CYC8* was deleted. We did not observe uniform derepression when Tup1 and/or Cyc8 sumoylation was disrupted (S1C Fig). However, the gene expression changes in this subset of genes was also distinct in Tup1^K270R^ cells compared with Cyc8^4KtoR^ cells, supporting that loss of Tup1 and Cyc8 sumoylation confer different outcomes.

We next examined gene expression changes during hyperosmotic stress (Fig 5B and S2 Table). To identify genes whose expression was altered in Tup1 and Cyc8 sumoylation-deficient strains relative to wild-type strains, we calculated mutant-effect ratios for each time point by subtracting the log_2_ values of the wild-type strain from each mutant strain [40,41]. We present those genes whose expression was changed by at least 1.57 fold in the mutant strains compared to the wild-type strain. Once again, Tup1^K270R^ cells showed a distinct pattern of gene expression changes when compared with Cyc8^4KtoR^ cells or Tup1^K270R^/Cyc8^4KtoR^ cells (Fig 5B). Focusing on the 16 example genes reported in other studies, we again did not observe uniform derepression when Tup1 and/or Cyc8 sumoylation was disrupted (S1D Fig), but gene expression changes in this subset of genes was also distinct between Tup1^K270R^ cells compared to Cyc8^4KtoR^ cells. All data thus far indicated that loss of Tup1 sumoylation has effects that are distinct from loss of Cyc8 sumoylation.

We inquired if there was any functional enrichment among the genes that showed altered expression in Tup1^K270R^ or Cyc8^4KtoR^ cells during hyperosmotic stress. To be stringent, we examined the dataset for genes with at least 2-fold expression changes in either Tup1^K270R^ or Cyc8^4KtoR^ cells 30 minutes after hyperosmotic stress. In Tup1^K270R^ cells, we discovered that 15 of the 16 upregulated genes are proximal to telomeres (S1E Fig), suggesting Tup1 sumoylation might play a role in regulating subtelomeric genes during hyperosmotic stress. In Cyc8^4KtoR^ cells, we found that 13 of the 21 upregulated genes are normally glucose repressed (S1F Fig), and some of these are hexose sugar transporters (*HXT1*, *HXT6*, and *HXT7*) that are known to be reduced in abundance during hyperosmotic stress [42]. These results are interesting because hexose sugars can also serve as intracellular osmolytes and the misregulation of hexose transporters and metabolic enzymes could contribute to delays in recovery from hyperosmotic stress. We confirmed the altered expression of two of these glucose-repressed genes, *HXT6* and *GSY1*, by quantitative RT-PCR (S1G Fig).

### Loss of Cyc8 sumoylation has subtle growth defects that are not fully penetrant

Because sumoylation-deficient Tup1^K270R^ cells showed distinct transcriptional changes from sumoylation-deficient Cyc8^4KtoR^ cells, we examined whether there were different phenotypic growth consequences of eliminating Tup1 or Cyc8 sumoylation. In unstressed conditions, we did not observe any growth differences between wild-type and mutant strains (Fig 5C and 5D). Using very stringent hyperosmotic stress conditions (1M NaCl), we did observe subtle growth defects for strains that were deficient for Cyc8 sumoylation (Fig 5C and 5D). However, we note that this phenotype was not fully penetrant; we reproducibly found that ~50% of cultures showed little effect (Fig 5C). This unusual and intriguing variability in phenotype suggested that there may be an underlying epigenetic effect associated with loss of Cyc8 sumoylation.

### Cyc8 sumoylation prevents inclusion formation of Cyc8-Tup1 during hyperosmotic stress

We were intrigued by the epistatic nature of Cyc8 sumoylation seen in the transcript array analysis. Specifically, we wanted to explore the mechanism by which loss of Cyc8 sumoylation overrides loss of Tup1 sumoylation. Sumoylation is generally thought to alter protein activity, localization, and/or interactions [3,14]. However, another possible role for sumoylation may be to protect against inclusion formation of misfolded proteins *in vivo* [43,44,45,46]. In fact, a key biochemical feature of SUMO is that it is a highly soluble protein and can promote the solubility of recombinant proteins when fused to them [47,48].

It has been reported that hyperosmotic stress induces protein misfolding *in vivo* [49,50], particularly of glutamine-rich proteins [51]. Cyc8 has a 16-residue polyglutamine tract from residues 15–30 and a highly glutamine-rich region from residues 491–598 (S2A Fig). The Cyc8 glutamine-rich region includes a 32 glutamine-alanine repeat sequence and a 31-residue polyglutamine tract that approaches the pathological length size linked to human polyglutamine-expansion diseases [52]. Interestingly, overexpression of a C-terminal fragment containing Cyc8’s glutamine-rich region (residues 465–966) induces the formation of a Cyc8 prion state—the yeast [OCT+] prion [35]. Consistent with its ability to form the [OCT+] prion, Cyc8 has been predicted to contain a region from residues 441–677 that has sequence properties of a prion domain (PrD) [53], and this includes the glutamine-rich region. In fact, overexpression of Cyc8’s predicted PrD leads to the formation of detergent-insoluble amyloids *in vivo* [53]. Tup1 also has 2 glutamine-rich regions from residues 97–128 and 181–202 (S2B Fig). Based on this information, we hypothesized that sumoylation might protect Cyc8 and possibly Tup1 from forming inclusions *in vivo* during hyperosmotic stress.

**Fig 6:**
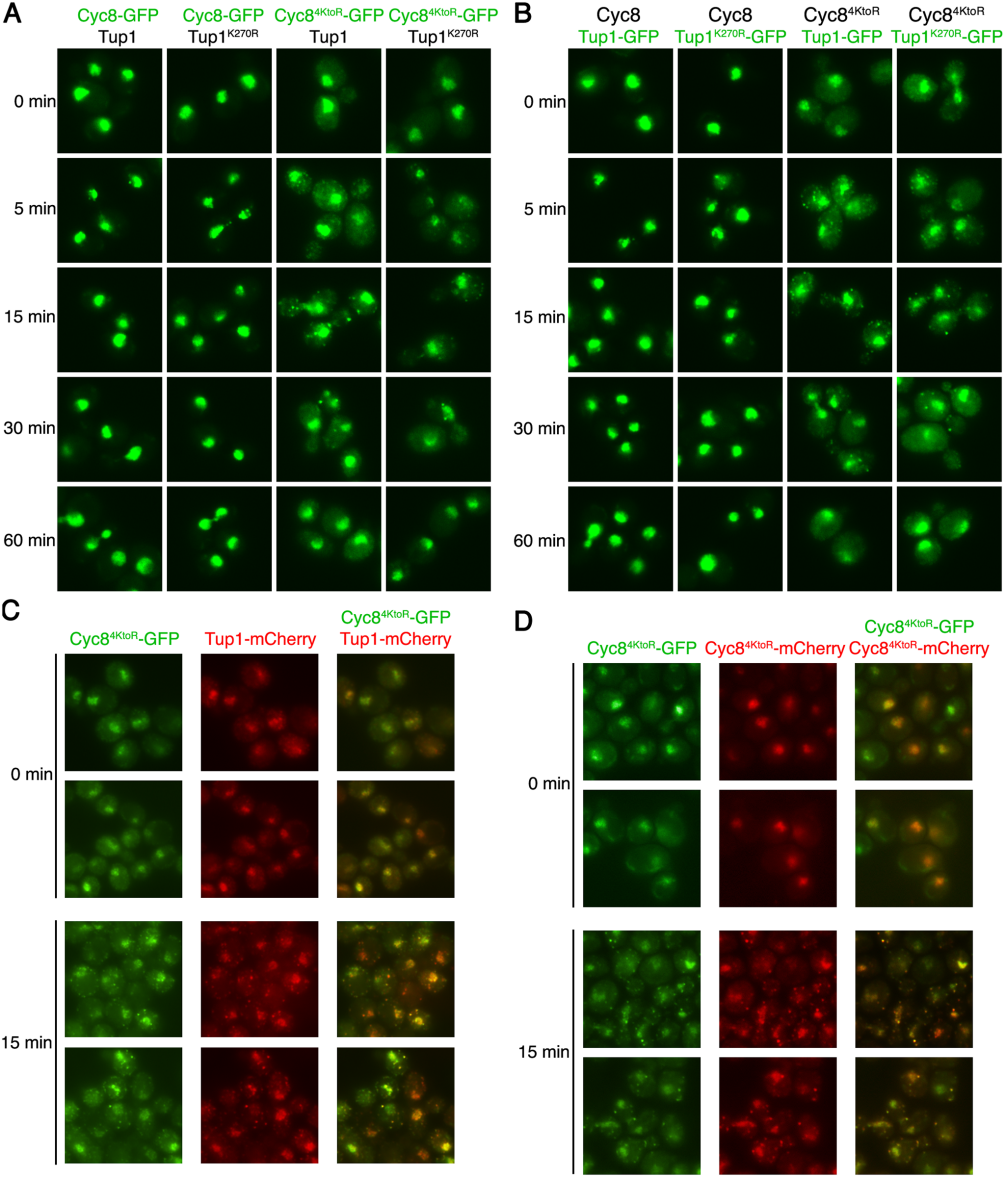
Loss of Cyc8 sumoylation leads to cytoplasmic inclusion formation of both Cyc8 and Tup1. (**A, B**) Strains expressing combinations of wild-type and/or sumoylation-deficient mutant versions of Cyc8 and Tup1, tagged with GFP or untagged, were exposed to a time course of hyperosmotic stress (1.2M sorbitol), fixed at the times indicated, and imaged by fluorescence microscopy. All constructs were integrated at the gene’s endogenous locus and expressed from the endogenous promoter. (A) GFP fluorescence images of cells in which wild-type or mutant Cyc8 is GFP-tagged and wild-type or mutant Tup1 is untagged. (B) GFP fluorescence images of cells in which wild-type or mutant Cyc8 is untagged and wild-type or mutant Tup1 is GFP-tagged. (**C**) GFP and mCherry images in which Cyc8^4KtoR^ is tagged with GFP and wild-type Tup1 is tagged with mCherry at their endogenous genomic loci. Cells were exposed to 0 or 15 minutes of hyperosmotic stress (1.2M sorbitol), fixed, and imaged by fluorescence microscopy. (**D**) GFP and mCherry images in which one copy of Cyc8^4KtoR^ is tagged with GFP at the endogenous genomic locus and another CEN plasmid-expressed copy is tagged with mCherry. Cells were exposed to 0 or 15 minutes of hyperosmotic stress (1.2M sorbitol), fixed, and imaged by fluorescence microscopy.

To examine inclusion formation for Cyc8, we made strains expressing GFP-tagged wild-type Cyc8 or sumoylation-deficient Cyc8^4KtoR^ in combination with untagged wild-type Tup1 or sumoylation-deficient Tup1^K270R^. After exposure of cells to hyperosmotic stress during a time course of 60 minutes, sumoylation-deficient Cyc8^4KtoR^-GFP, but not wild-type Cyc8-GFP, formed transient cytoplasmic inclusions that peaked in number during the first 15 minutes of hyperosmotic stress and disappeared by 60 minutes (Fig 6A). The inclusions formed by Cyc8^4KtoR^-GFP were independent of Tup1 sumoylation as the inclusions formed in cells with either wild-type Tup1 or sumoylation-deficient Tup1^K270R^.

We next examined if sumoylation-deficient Tup1^K270R^ also formed inclusions during hyperosmotic stress. We generated strains expressing GFP-tagged wild-type Tup1 or sumoylation-deficient Tup1^K270R^ in combination with untagged wild-type Cyc8 or sumoylation-deficient Cyc8^4KtoR^. Neither Tup1-GFP nor Tup1^K270R^-GFP formed inclusions over the course of hyperosmotic stress in wild-type Cyc8 cells (Fig 6B). However, both Tup1-GFP and Tup1^K270R^-GFP formed transient inclusions in sumoylation-deficient Cyc8^4KtoR^ cells, peaking at 15 minutes and disappearing by 60 minutes. Thus, the loss of Cyc8 sumoylation caused both Cyc8 and Tup1 to form transient cytoplasmic inclusions during hyperosmotic stress. This also explains why loss of Cyc8 sumoylation is epistatic to loss of Tup1 sumoylation.

Because both Cyc8 and Tup1 formed inclusions when Cyc8 was sumoylation deficient, we explored whether the individual Tup1 and Cyc8 inclusions were due to the Cyc8-Tup1 complex forming inclusions. To do this, we tagged Cyc8^4KtoR^ with GFP and Tup1 with mCherry to determine if Cyc8^4KtoR^ and Tup1 inclusions colocalize in the same cell. After addition of 1.2M sorbitol to induce hyperosmotic stress, we observed that Tup1-mCherry inclusions did colocalize with Cyc8^4KtoR^-GFP inclusions (Fig 6C), indicating that the inclusions are Tup1-Cyc8 complexes.

We also determined if the inclusions were composed of multiple Cyc8^4KtoR^ molecules. To do this, we coexpressed Cyc8^4KtoR^ tagged with GFP and Cyc8^4KtoR^ tagged with mCherry in the same cells. After induction of hyperosmotic stress, we found that both Cyc8^4KtoR^-GFP and Cyc8^4KtoR^-mCherry colocalized to the same inclusions (Fig 6D). This indicates that the inclusions contain multiple Cyc8 molecules.

**Fig 7:**
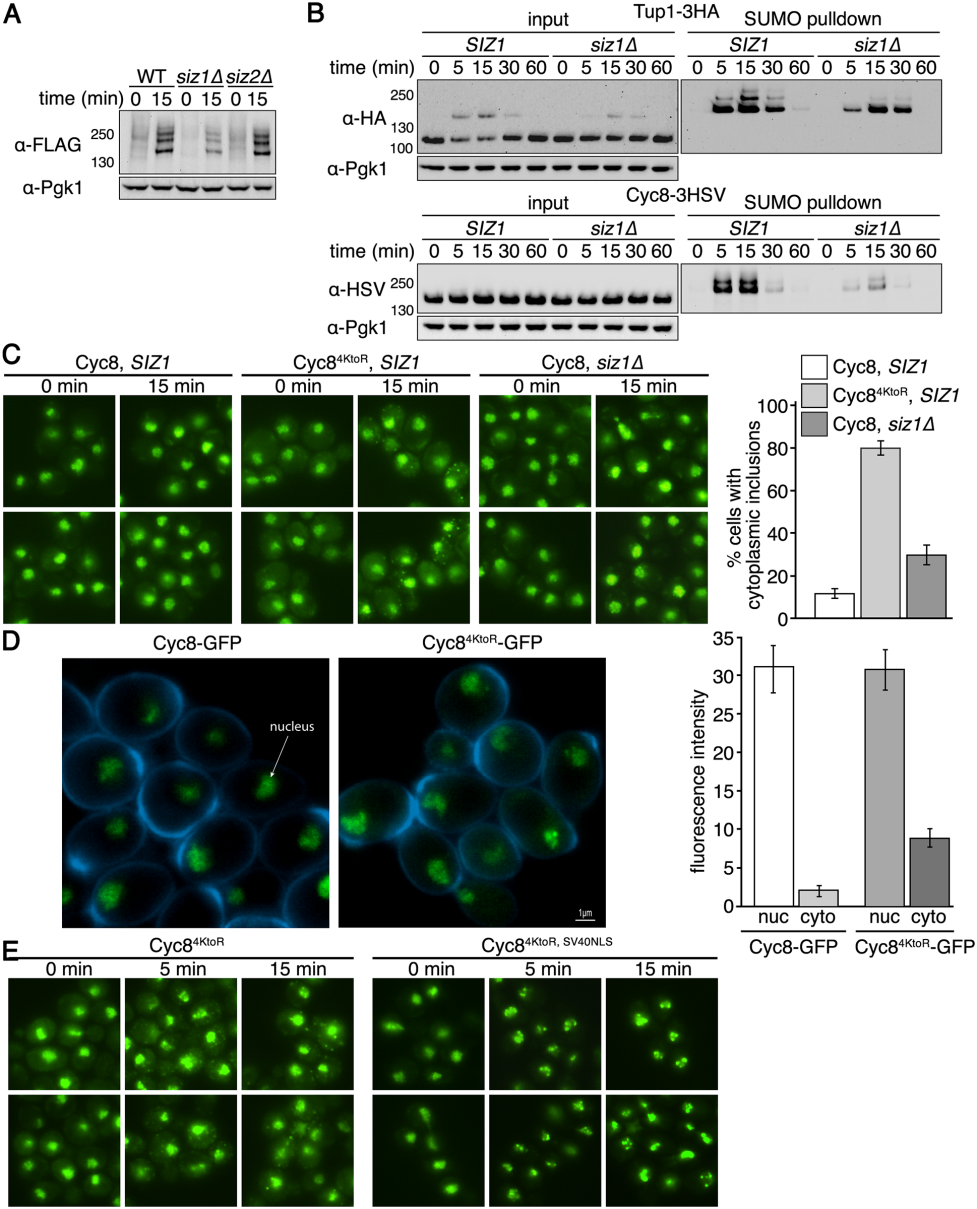
Siz1 is a SUMO ligase that influences Cyc8 sumoylation. (**A**) *His*_*6*_*-FLAG-SMT3* wild-type, *siz1*Δ, or *siz2*Δ cells were subject to hyperosmotic stress (1.2M sorbitol) for 0 or 15 minutes. Global sumoylation patterns were examined by western analysis using an anti-FLAG antibody. (**B**) *His*_*6*_*-FLAG-SMT3* wild-type or *siz1*Δ cells expressing either Tup1-3HA or Cyc8-3HSV from their endogenous promoters were subject to hyperosmotic stress (1.2M sorbitol) over a 60-minute time course. Cell lysates were generated and subject to metal affinity chromatography to purify sumoylated proteins. Cell lysates (input) and purified sumoylated proteins (SUMO pulldown) were subject to SDS-PAGE and western analyses using anti-HA (Tup1) or anti-HSV (Cyc8) antibodies. (**C**) *SIZ1* or *siz1*Δ cells expressing wild-type Cyc8 or sumoylation-deficient Cyc8^4KtoR^ tagged with GFP from the endogenous *CYC8* promoter were exposed to hyperosmotic stress (1.2M sorbitol) for 0 and 15 minutes, fixed at the times indicated, and imaged by fluorescence microscopy. Six fields of cells for each condition, with ≥40 cells/field, were counted for the presence of cytoplasmic inclusions. Data represent the average percentage of cells that contained cytoplasmic inclusions within the six fields. Error bars are the standard deviation. (**D**) Confocal 3D images and quantitation of Cyc8-GFP and Cyc8^4KtoR^-GFP localization in the nucleus and cytoplasm. The nuclear and cytoplasmic fluorescence intensities were measured using NIS-Elements software (Nikon). Data represents the average of at least 100 cells in at least 10 fields in 3 independent experiments. Error bars represent the standard deviation. (**E**) Cells expressing GFP-tagged versions of Cyc8^4KtoR^ or Cyc8^4KtoR^ with the SV40 NLS appended to the C-terminus (both expressed from the endogenous *CYC8* promoter) were exposed to hyperosmotic stress (1.2M sorbitol) for 0, 5, and 15 minutes, fixed at the times indicated, and imaged by fluorescence microscopy.

One possibility for the inclusion formation seen in Cyc8^4KtoR^ cells is that the *in cis* mutations within Cyc8 that eliminate sumoylation cause Cyc8 to become more prone to inclusion formation irrespective of sumoylation. Previously, it was found that the SUMO ligase Siz1 is primarily involved in the global sumoylation observed during hyperosmotic stress [13,21]. Therefore we made a strain with *SIZ1* deleted, and verified that loss of Siz1 function reduced global sumoylation during hyperosmotic stress whereas loss of Siz2 function did not (Fig 7A). We then explored the effect of losing Siz1 function on Tup1 and Cyc8 sumoylation. In *siz1*Δ cells, Tup1 sumoylation was reduced but still considerable (Fig 7B). By contrast, Cyc8 sumoylation was more greatly reduced in *siz1*Δ cells (Fig 7B), indicating that we were able to differentially reduce Cyc8 sumoylation in *siz1*Δ cells. When we examined inclusion formation, we observed that wild-type Cyc8 in *siz1*Δ cells formed similar inclusions as Cyc8^4KtoR^ in *SIZ1* cells, but at a lower frequency (Fig 7C). Thus, loss of sumoylation *in cis* or *in trans* leads to persistent Cyc8 inclusion formation during hyperosmotic stress.

We observed that Cyc8^4KtoR^ exhibited a modest increase in cytoplasmic localization by fluorescence microscopy. One possible reason for this is that mutation of Cyc8’s sumoylation sites alters its nuclear import. Therefore, we quantified the amount of Cyc8 present in the nucleus and cytoplasm in cells expressing wild-type Cyc8 or sumoylation-deficient Cyc8^4KtoR^. We did not observe a change in the nuclear intensity of Cyc8 in either cells (Fig 7D), indicating that nuclear import is not compromised by mutation of Cyc8’s sumoylation sites. It is possible that the increased cytoplasmic localization of Cyc8^4KtoR^ occurs because mutating the four lysine residues alters cytoplasmic protein quality control, potentially at the ribosome [54]. To determine what would happen when the Cyc8^4KtoR^ cytoplasmic pool was redirected to the nucleus, we added the strong SV40 NLS to Cyc8^4KtoR^. This caused Cyc8^4KtoR, SV40NLS^ to become predominantly nuclear localized (Fig 7E). More importantly, the inclusion behavior was unchanged and we observed visually dramatic inclusion formation in the nucleus with Cyc8^4KtoR, SV40NLS^ (Fig 7E). We conclude that the behavior of the protein is unchanged whether in the nucleus or cytoplasm.

### Sumoylation-deficient Cyc8 cytoplasmic inclusions are not P bodies or stress granules

**Fig 8:**
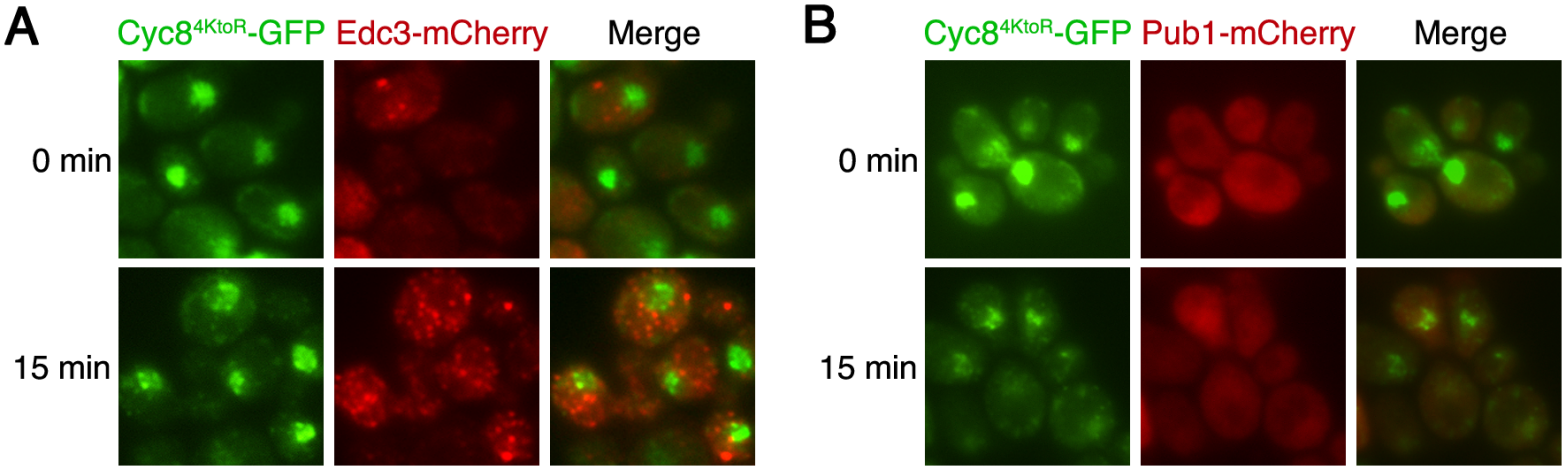
Cytoplasmic Cyc8^4KtoR^-GFP inclusions are not P bodies or stress foci. Cells expressing sumoylation-deficient Cyc8^4KtoR^ tagged with GFP from the endogenous *CYC8* promoter or (**A**) Edc3 tagged with mCherry from the endogenous *EDC3* promoter or (**B**) Pub1 tagged with mCherry from the endogenous *PUB1* promoter were exposed to hyperosmotic stress (1.2M sorbitol) for 0 and 15 minutes, fixed, and imaged by fluorescence microscopy.

P bodies and stress granules are dynamic cellular inclusions that form when translation initiation is compromised [55]. Because hyperosmotic stress challenges the cell’s translational capacity, we explored if the sumoylation-deficient Cyc8^4KtoR^ cytoplasmic inclusions were P bodies and/or stress granules. The protein Edc3 is involved in mRNA decapping and forms P bodies during glucose deprivation, oxidative stress, and hyperosmotic stress [55]. The protein Pub1 is a poly(A) RNA-binding protein that stabilizes many mRNAs and forms stress granules during glucose deprivation and oxidative stress [55]. To examine P bodies and stress granule formation, we tagged Edc3 or Pub1 with mCherry in cells that also expressed sumoylation-deficient Cyc8^4KtoR^ tagged with GFP. In no case did we observe colocalization of Cyc8^4KtoR^ inclusions with Edc3 P bodies that formed under hyperosmotic stress (Fig 8A). Pub1 stress granules do not form under hyperosmotic stress ([55] and Fig 8B), thus eliminating the possibility that sumoylation-deficient Cyc8^4KtoR^ inclusions are stress granules.

### Normal Cyc8 and sumoylation-deficient Cyc8 form nuclear inclusions during hyperosmotic stress with different resolution kinetics

**Fig 9:**
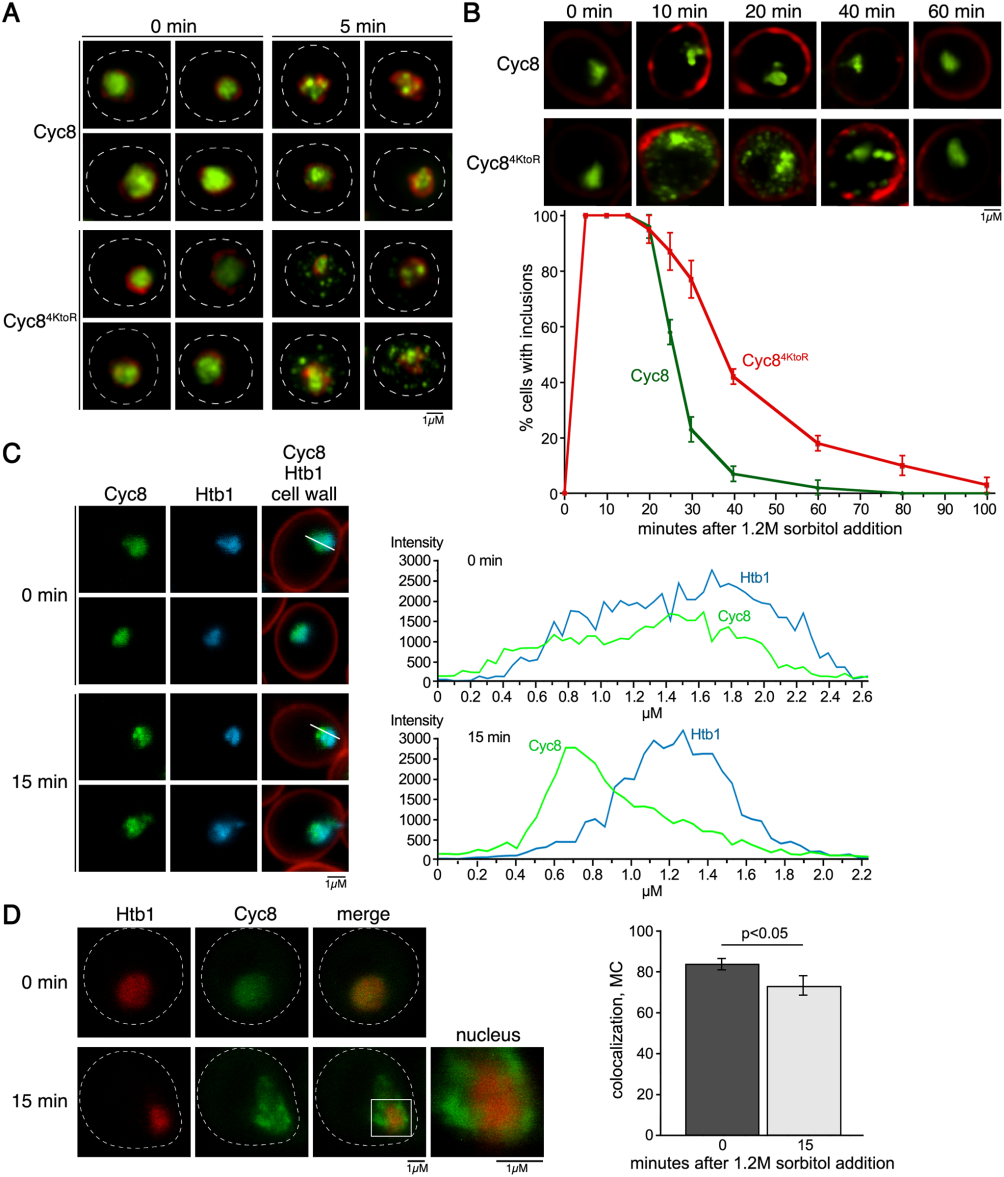
Cyc8 inclusions form in the nucleus during hyperosmotic stress. (**A**) Structured Illumination Microscopy (SIM) images of cells subjected to 0 or 5 minutes of hyperosmotic stress (1.2M sorbitol). Wild-type or mutant Cyc8 is GFP-tagged and Nup53 is mCherry-tagged. (**B**) SIM images of cells during a 60 minute time course of hyperosmotic stress (1.2M sorbitol). Wild-type or mutant Cyc8 is GFP-tagged, and the cell wall is stained with Calcofluor White. Graph shows the percentage of cells with inclusions over time. (**C**) Confocal 3D images of cells subjected to 0 or 15 minutes of hyperosmotic stress (1.2M sorbitol). Wild-type Cyc8 is GFP-tagged and histone H2B (Htb1) is mCherry-tagged. Graph shows the intensity of Cyc8 or Htb1 across the white line drawn on the cells to the left. (**D**) Confocal 3D images of cells expressing Cyc8-GFP or Htb1-mCherry. Graph to the right shows the Mander's overlap coefficient [56,57] for the colocalization of the proteins during 0 and 15 minutes of hyperosmotic stress (1.2M sorbitol).

Wild-type Cyc8 and sumoylation-deficient Cyc8^4KtoR^ are both highly enriched in the nucleus (Fig 6A), which is expected for Cyc8’s transcription corepressor function. However, mutant Cyc8^4KtoR^ showed some cytoplasmic localization compared to wild-type Cyc8 (Fig 6A). Furthermore, Cyc8^4KtoR^ inclusions were most evident in the cytoplasm by standard fluorescence microscopy (Fig 6A). To be thorough, we wanted to determine if the nuclear-localized fraction of sumoylation-deficient Cyc8^4KtoR^ also formed inclusions. To do this, we used Structured Illumination Microscopy (SIM) to observe nuclear structures at greater visual resolution than can be achieved with standard fluorence microscopy. Using this approach, we were able to visualize that sumoylation-deficient Cyc8^4KtoR^ formed transient nuclear inclusions in addition to cytoplasmic inclusions (Fig 9A and 9B). Thus, inclusion formation is not merely a consequence of the cytoplasmic localization of sumoylation-deficient Cyc8^4KtoR^. Interestingly, wild-type Cyc8 also formed nuclear inclusions (Fig 9A and 9B), but the wild-type Cyc8 inclusions disappeared faster than the sumoylation-deficient Cyc8^4KtoR^ nuclear inclusions (Fig 9B). Disappearance of wild-type Cyc8 inclusions occurred ~20 minutes after hyperosmotic stress (Fig 9B), which is also the time of peak Cyc8 sumoylation (Figs 2D and 7B). This is consistent with a role for sumoylation in protecting wild-type Cyc8 from persistent inclusions during hyperosmotic stress.

It is possible that the nuclear inclusions formed by Cyc8 are not aggregates but concentrated chromatin-associated foci involved in active transcription during hyperosmotic stress. To explore the active chromatin possibility, we tagged histone H2B (Htb1) with mCherry in cells with Cyc8 tagged with GFP. We chose histone H2B because it is a general reporter for the dynamics of chromatin. We expect that we would observe similar H2B nuclear foci if Cyc8 condenses into chromatin-associated foci. Although Cyc8 and H2B localization were highly correlated during unstressed conditions, after hyperosmotic stress the peak localizations of Cyc8 and H2B intensities were very different (Fig 9C). Furthermore, when we used Mander’s overlap coefficient to measure colocalization after confocal microscopy [56,57], we found that the colocalization of Cyc8 and H2B decreased during hyperosmotic stress and this correlated with the inclusion formation of Cyc8 (Fig 9D). This suggests that the nuclear Cyc8 inclusions are unlikely to be chromatin-associated foci involved in transcription.

### Loss of Hog1 function causes longer persistence of Cyc8 inclusions

Cellular resolution of sumoylation-deficient Cyc8^4KtoR^ inclusions did not occur until 40–60 minutes after application of hyperosmotic stress (Figs 6A and 9B). The observation that Cyc8^4KtoR^ inclusions resolved in the absence of Cyc8 sumoylation could be explained by how eukaryotic cells adapt to hyperosmotic stress [58]. Activation of the Hog1/p38 MAP kinase pathway allows cells to neutralize hyperosmotic stress by accumulating intracellular osmolytes and chemical chaperones, most importantly glycerol [59]. In yeast, intracellular glycerol accumulation peaks in concentration ~40–60 minutes after initiation of the hyperosmotic stress [60], and this is due to Hog1’s inactivation of glycerol efflux pumps and activation of glycerol synthesis enzymes [59].

**Fig 10:**
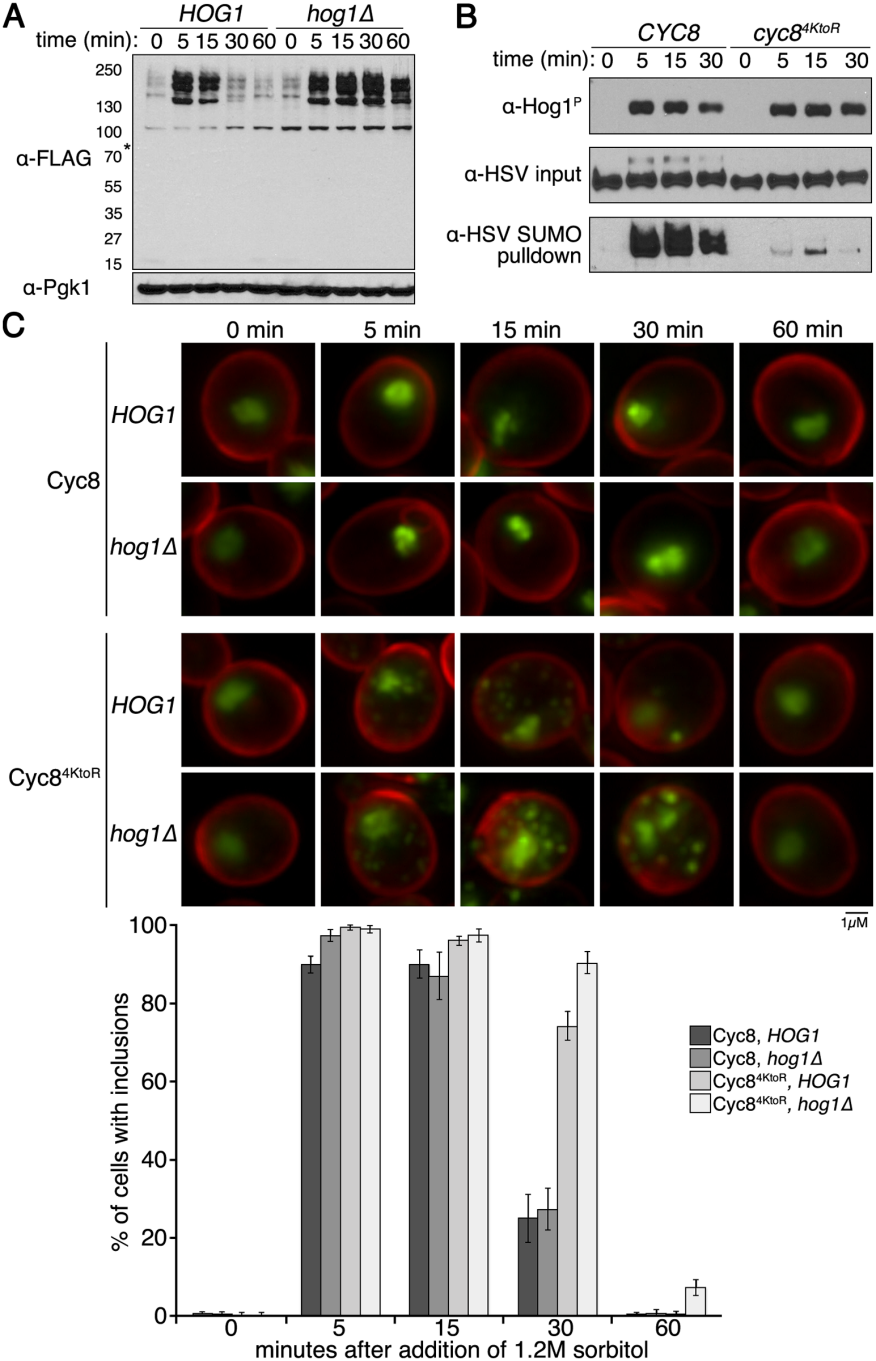
Hog1 pathway influences the duration of hyperosmotic stress induced sumoylation and Cyc8^4KtoR^ inclusions. (**A**) *His*_*6*_*-FLAG-SMT3* cells with the *HOG1* or *hog1*Δ allele were subjected to a time course of hyperosmotic stress (1.2M sorbitol). Changes in sumoylation patterns were examined by western analysis using an anti-FLAG antibody. (**B**) *His*_*6*_*-FLAG-SMT3* cells expressing either wild-type Cyc8 or mutant Cyc8^4KtoR^ were subjected to a time course of hyperosmotic stress (1.2M sorbitol). Phosphorylated Hog1 (top panel), wild-type Cyc8 and mutant Cyc8^4KtoR^ levels (middle panel), and sumoylated wild-type Cyc8 and mutant Cyc8^4KtoR^ levels (bottom panel) were examined by western analysis using an anti-phosphorylated Hog1 antibody and the HSV antibody that detects Cyc8-3HSV. (**C**) SIM images of *HOG1* or *hog1*Δ cells under a time course of hyperosmotic stress. Wild-type Cyc8 and mutant Cyc8^4KtoR^ are tagged with GFP, and the cell wall is stained with Calcofluor White. Quantitation of results is shown. The presence of inclusions was analyzed by counting the cells with at least one visible inclusions. At least 200 cells in at least 5 fields were analyzed for each time point. Data in the graph represent the average of 3 independent experiments. Error bars represent the standard deviation.

Therefore, we examined whether loss of Hog1 signaling altered hyperosmotic stress-induced sumoylation as well as sumoylation-deficient Cyc8^4KtoR^ inclusion formation/persistence. The kinetics of global hyperosmotic stress-dependent sumoylation were identical between *HOG1* and *hog1*Δ cells, but desumoylation was markedly slower in *hog1*Δ cells (Fig 10A). It is important to note that loss of Cyc8 sumoylation did not alter the kinetics of Hog1 phosphorylation and activation during hyperosmotic stress (Fig 10B), indicating that Hog1 activation is independent of Cyc8 sumoylation. Hog1 phosphorylation remains slightly higher in *cyc8*^*4KtoR*^ cells at 30 minutes of hyperosmotic stress (Fig 10B). This delay is consistent with an overall delayed adaptation to hyperosmotic stress response in *cyc8*^*4KtoR*^ cells, which can be observed as a growth defect of *cyc8*^*4KtoR*^ cells in hyperosmotic conditions (Fig 5C and 5D). In addition, sumoylation-deficient Cyc8^4KtoR^ inclusions formed with the same kinetics in both *HOG1* cells and *hog1*Δ cells, but were visually more pronounced and persisted much longer in *hog1*Δ cells (Fig 10C). We conclude that hyperosmotic stress-induced Cyc8 sumoylation and Cyc8^4KtoR^ inclusion formation do not require Hog1, but their persistence is modulated by Hog1 function.

### The glutamine-rich prion domain of Cyc8 mediates inclusion formation

**Fig 11:**
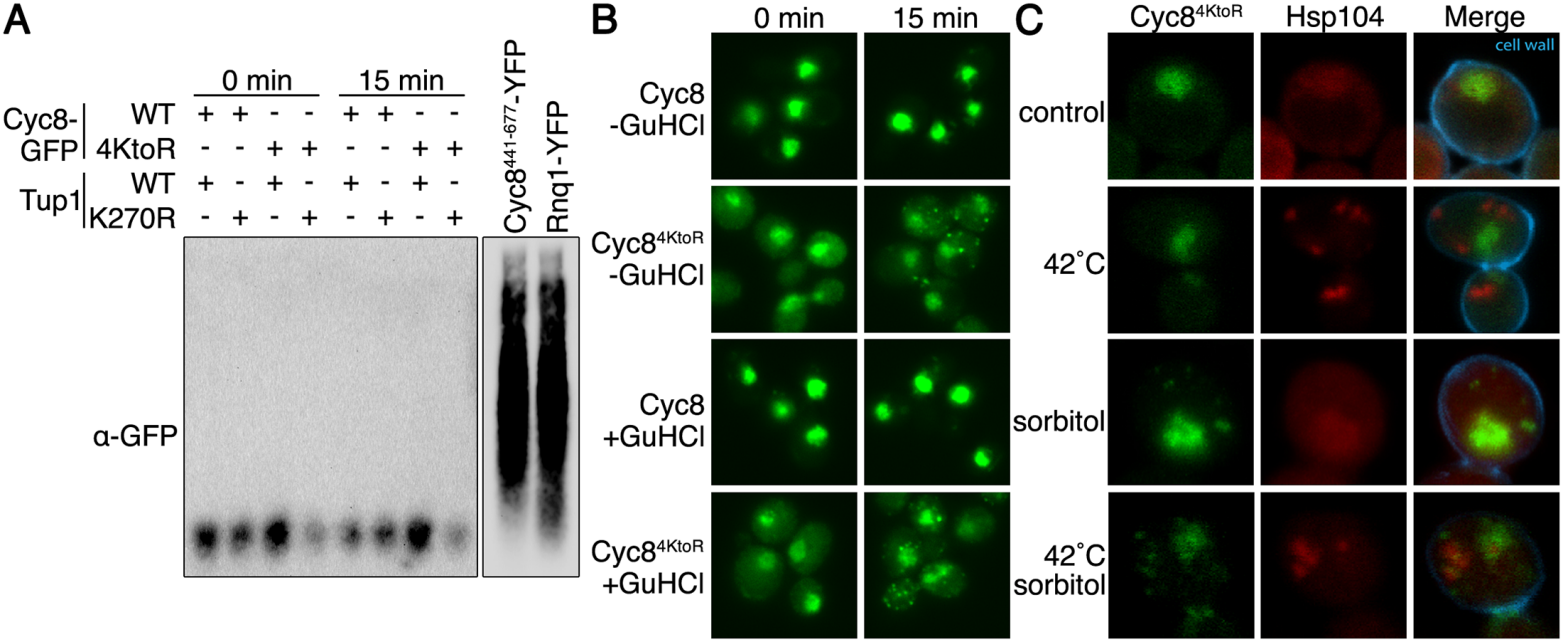
Cyc8 inclusions are not SDS-resistant, Hsp104-dependent amyloids. (**A**) Semi-denaturing detergent agarose gel electrophoresis (SDD-AGE) of lysates from the strains used in Fig 6A. Cells were exposed to 0 or 15 minutes of hyperosmotic stress (1.2M sorbitol), and lysates were mixed with semi-denaturing buffer before loading into the agarose gel. Lysates from strains overexpressing either Rnq1-YFP or Cyc8^441-677^-YFP are also included as positive controls for SDS-resistant amyloid formation. Western analysis was performed with anti-GFP antibody. The two images are different exposures of the same blot. (**B**) Fluorescence microscopy of cells expressing GFP-tagged wild-type or sumoylation-deficient Cyc8 after 3 passages on either YPD or YPD+4mM guanidinium hydrochloride (GuHCl). Cells were exposed to 0 or 15 minutes of hyperosmotic stress (1.2M sorbitol), fixed, and imaged by fluorescence microscopy. All constructs were integrated at the gene’s endogenous locus and expressed from the endogenous promoter. (**C**) Confocal 3D images of cells expressing mutant Cyc8^4KtoR^ tagged with GFP and Hsp104 tagged with mCherry were subject to a 15 minute 42°C heat shock to induce Hsp104 inclusions, 15 minute hyperosmotic stress (1.2M sorbitol) to induce Cyc8^4KtoR^ inclusions, or both. Cell wall was visualized by staining with Calcofluor White.

Cyc8 has a glutamine-rich prion domain (PrD) that, when overexpressed by itself, can induce formation of the Cyc8 [OCT+] prion [35], and form SDS-resistant amyloids [53]. Because we examined full-length Cyc8 under endogenous expression conditions, we wanted to determine whether the hyperosmotic stress-dependent Cyc8^4KtoR^-GFP inclusions adopt an SDS-resistant amyloid state. One common technique to assess the amyloid nature of yeast prions is semi-denaturing detergent agarose gel electrophoresis (SDD-AGE) [53,61,62]. Using SDD-AGE, we found Cyc8^4KtoR^-GFP did not form SDS-resistant amyloids during hyperosmotic stress (Fig 11A). As controls, overexpression of the Cyc8 PrD by itself or the known prion Rnq1 did form SDS-resistant amyloids (Fig 11A). In a second comparison to amyloid prions, we found that Cyc8^4KtoR^-GFP still formed identical inclusions after passaging cells on medium containing guanidinium hydrochloride (GuHCl) (Fig 11B), which is a treatment that suppresses the propagation of some amyloidogenic prion proteins in yeast by inhibiting Hsp104 function [63]. Furthermore, sumoylation-deficient Cyc8^4KtoR^ cytoplasmic inclusions did not colocalize with heat-induced Hsp104 inclusions (Fig 11C), supporting the idea that the formation of Cyc8^4KtoR^ inclusions is independent of Hsp104 function. These data indicate that transient Cyc8^4KtoR^-GFP inclusions have not achieved an Hsp104-dependent amyloid state during hyperosmotic stress.

**Fig 12:**
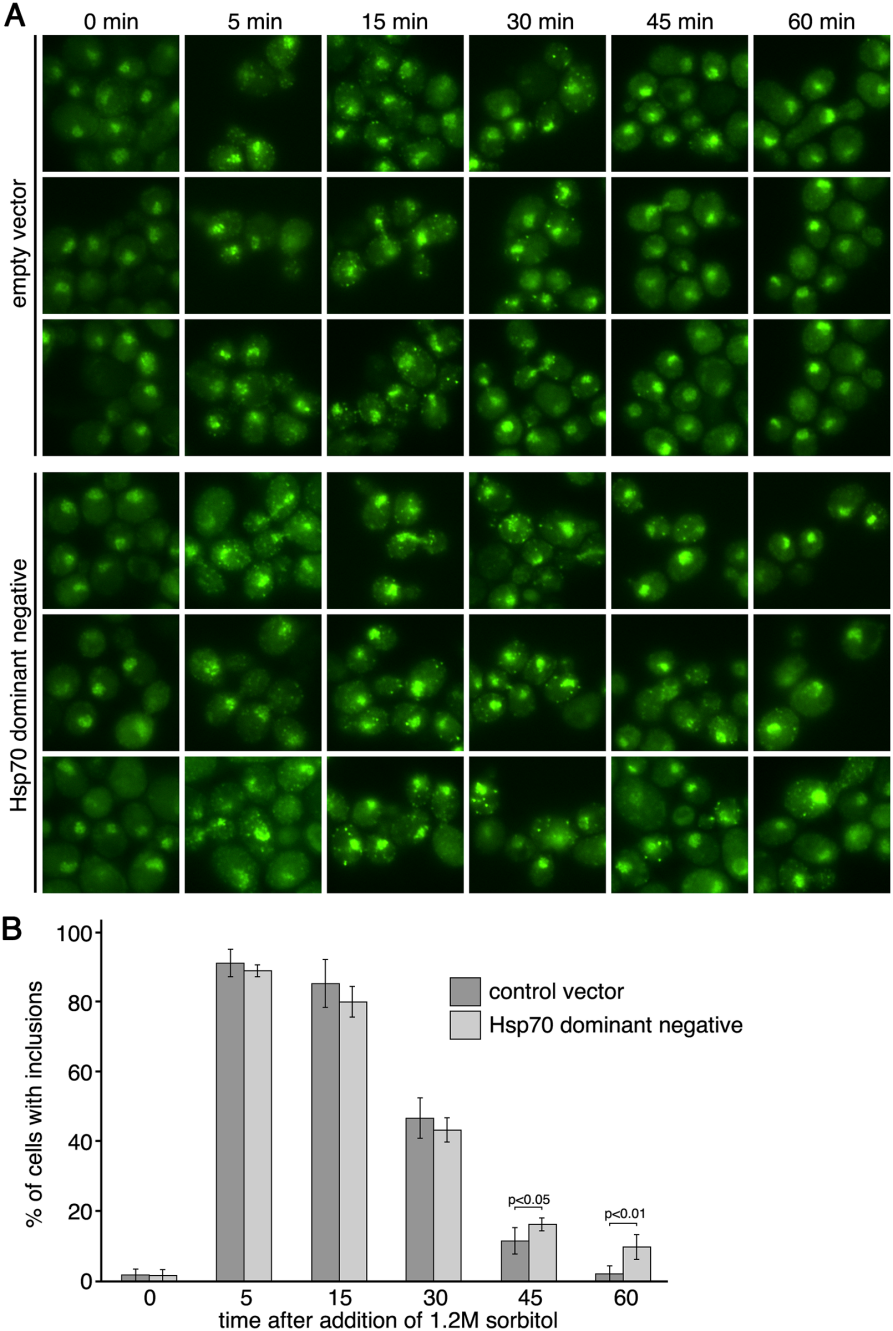
Expression of a dominant-negative form of Hsp70 influences the persistence of Cyc8^4KtoR^ inclusions. (**A**) Cells expressing Cyc8^4KtoR^-GFP from the endogenous *CYC8* locus and carrying either an empty 2 micron vector or a 2 micron vector with an dominant-negative form of Hsp70 (*SSA1*) were exposed to a time course of hyperosmotic stress (1.2M sorbitol), fixed at the times indicated, and imaged by fluorescence microscopy. (**B**) Analysis of data from (A). Six fields of cells for each condition, with at least 40 cells/field, were counted for the presence of cytoplasmic inclusions. Data represent the average percentage of cells that contained cytoplasmic inclusions within the six fields. Error bars are the standard deviation. A two-tailed, heteroscedastic student’s t-test was used to determine significance.

Some prionogenic proteins have been found to form non-amyloid cellular inclusions that are independent of Hsp104 function but dependent upon the activity of Hsp70 [64,65]. These studies revealed the importance of Hsp70 by overexpression of a dominant-negative inactive form of Hsp70. Therefore, we induced hyperosmotic stress and examined Cyc8^4KtoR^ inclusion formation in cells expressing a dominant-negative inactive version of Hsp70 from a 2 micron plasmid [64,65]. We found that this condition influenced the persistence of Cyc8 inclusions, with the dominant-negative inactive Hsp70 strain exhibiting more pronounced and persistent inclusions at 45 and 60 minutes of hyperosmotic stress (Fig 12A and 12B).

Protein inclusions exist on a spectrum within cells ranging from highly unstable, dynamic structures to extremely stable, static structures such as amyloids [66,67,68]. Although the sumoylation-deficient Cyc8^4KtoR^-GFP inclusions did not form amyloids, we assessed if they could form other stable aggregates using several biochemical approaches, including fractionation of cell lysates to assess insolubility and ultracentrifugation to query for smaller oligomers. In no case could we observe the persistence of stable aggregates that survived the cell lysis procedures used for the biochemical assays. These results suggest that, under the biologically relevant conditions of our experiments in which endogenously expressed sumoylation-deficient Cyc8^4KtoR^ transiently forms and then disperses from inclusions *in vivo*, the inclusions are not stable enough to survive the lysis and analysis conditions often used to assess stable aggregate formation biochemically *in vitro*. In light of these observations, we think it is important to note that the vast majority of studies examining cellular aggregation/inclusion formation use massive overexpression of the misfolded proteins. This makes analyses much more facile because the overexpressed proteins form relatively stable inclusions that can be assessed for their insolubility/aggregation by bulk biochemical assays. In thinking about lower abundance endogenously expressed proteins, the dynamic view of self-association and aggregation is likely the better way to frame the behavior of aggregation-prone proteins [66,67].

**Fig 13:**
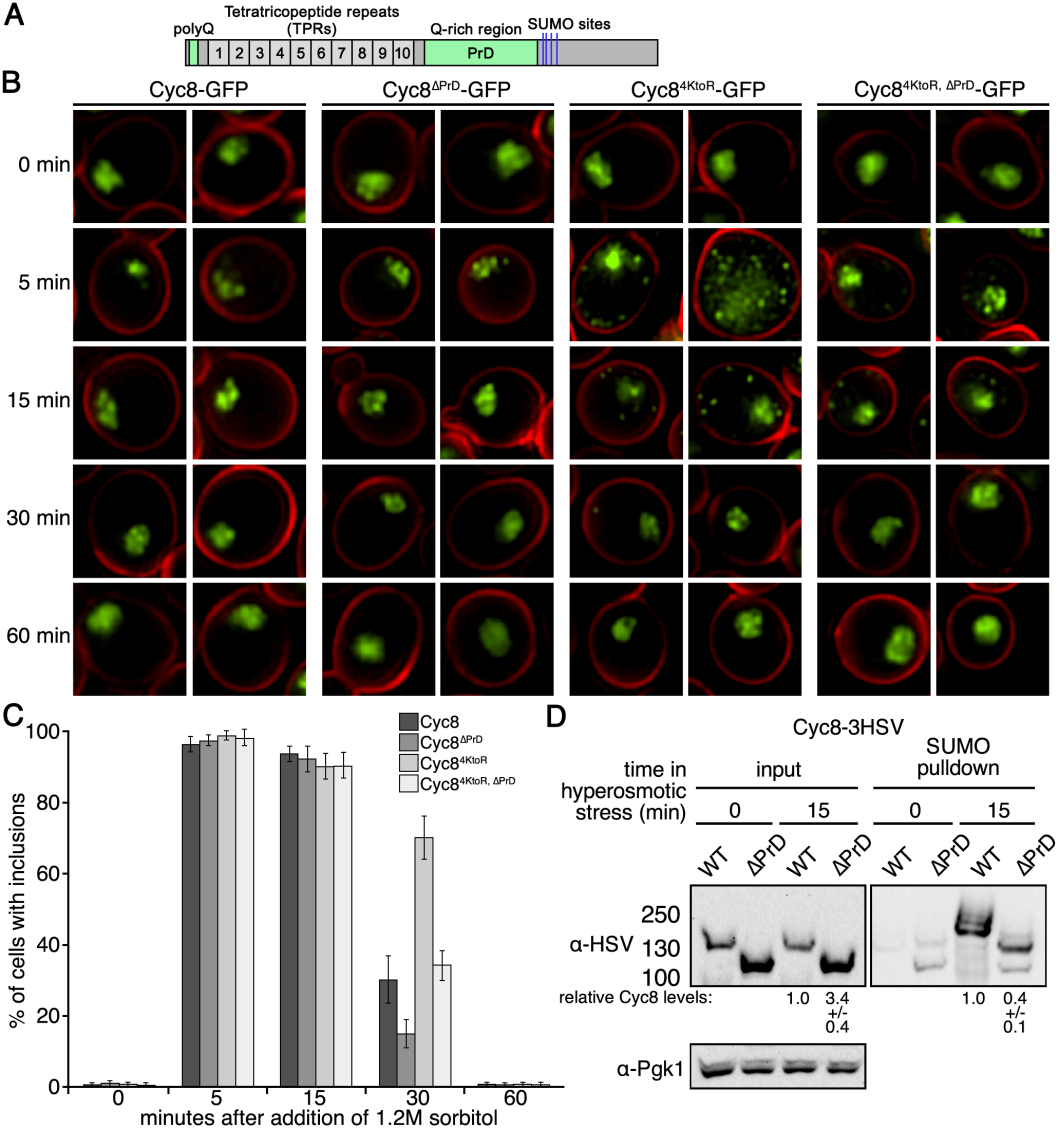
The Cyc8 prion domain (PrD) contributes to inclusion formation in the absence of sumoylation. (**A**) Domain schematic of Cyc8 with the polyglutamine tract and glutamine-rich PrD (residues 441–677) are shown in green, and the sumoylation sites identified in this study are shown in blue. (**B**) SIM images of cells expressing GFP-tagged, wild-type Cyc8 or sumoylation-deficient Cyc8^4KtoR^ with or without the PrD. Cells were exposed to hyperosmotic stress (1.2M sorbitol) for the indicated times. All constructs were integrated at the gene’s endogenous locus and expressed from the endogenous promoter. (**C**) Analysis of data from (B). The presence of inclusions was analyzed by counting the cells with at least one inclusion. At least 200 cells in at least 5 fields were analyzed for each time point. Data in the graph represent the average of 3 independent experiments. Error bars represent the standard deviation. (**D**) *6His-FLAG-SMT3* cells expressing wild-type Cyc8-3HSV or Cyc8^ΔPrD^-3HSV were examined for Cyc8 sumoylation at 0 or 15 minutes of hyperosmotic stress (1.2M sorbitol). Cell lysates (input) and purified sumoylated proteins (SUMO pulldown) were subject to western analyses using anti-HSV antibodies to detect Cyc8. Relative levels of the Cyc8^ΔPrD^ protein compared with the full-length Cyc8 protein are indicated below the input and SUMO pulldown lanes.

Using a cell biological approach, we wanted to investigate potential drivers of the transient inclusion formation of sumoylation-deficient Cyc8. We hypothesized that the glutamine-rich PrD was involved in the transient formation of Cyc8^4KtoR^ inclusions during hyperosmotic stress. The Cyc8 PrD spans residues 441–677 ([53]; Fig 13A and S2A Fig). We deleted the PrD in both wild-type Cyc8 and sumoylation deficient Cyc8^4KtoR^. We then expressed the PrD intact or deleted forms as the only version of Cyc8 in cells, and examined the presence of inclusions at 0, 5, 15, 30 and 60 minutes after initiation of hyperosmotic stress. The number of cells with at least one inclusion at 30 minutes was reduced by ~50% in Cyc8^ΔPrD^-GFP and Cyc8^4KtoR, ΔPrD^-GFP cells compared with Cyc8-GFP and Cyc8^4KtoR^-GFP cells (Fig 13B and 13C). From these results, we conclude that the Cyc8 PrD is important, but not essential for the formation and persistence of inclusions. Other regions that could participate in the initial stages of sumoylation-deficient Cyc8 inclusion formation include the N-terminal 16-residue polyglutamine tract in Cyc8 (Fig 13A and S2A Fig), the highly disordered C-terminal region of Cyc8 that follows the PrD (S2A Fig), the two glutamine-rich regions in Tup1 (S2B Fig), or the highly disordered N-terminal region of Tup1 that spans residues 100–300 (S2B Fig). Future work will be required to assess the contributions of these different domains on the kinetics of Cyc8 inclusion formation during hyperosmotic stress.

Lastly, we examined the levels of Cyc8 sumoylation with and without an intact PrD. After normalizing to the amount of Cyc8 input protein, we found there was a reduction in Cyc8 sumoylation of ~12 fold when the PrD was deleted (Fig 13D). Furthermore, it appears that the sumoylation laddering is dramatically reduced in Cyc8^ΔPrD^. Thus, the hyperosmotic stress-dependent sumoylation of Cyc8 is sensitive to the presence of the PrD.

## Discussion

In this work, we identified Tup1 and Cyc8 as major proteins that are dynamically sumoylated during hyperosmotic stress. Microarray analyses revealed that loss of Tup1 and Cyc8 sumoylation each conferred distinct outcomes on gene expression. Although we currently don’t understand the function of Tup1 sumoylation, gene expression changes suggest that sumoylation of Tup1 might play a role in regulating expression of telomere-proximal genes during hyperosmotic stress. We find this interesting due to computational evidence that supports a function for Tup1 in regulating telomere-associated gene clusters [69]. Conversely, loss of Cyc8 sumoylation appears to have more widespread expression changes during hyperosmotic stress. Through cell biological experiments, we discovered that Cyc8 sumoylation prevents the persistent formation of Cyc8-Tup1 inclusions after initiation of hyperosmotic stress. Because Cyc8’s prion-forming domain (PrD) is important for hyperosmotic stress-induced inclusion persistence, we propose that sumoylation of Cyc8 acts, in part, as a post-translational factor to prevent the persistence of Cyc8 inclusions during conditions of hyperosmotic stress.

### Transcriptional modulation through Cyc8 and Tup1 sumoylation

Previous studies have revealed a role for SUMO in modulating gene induction. Specifically, SUMO has been shown to accumulate at promoters during activation of inducible genes [70]. In addition, reduction of SUMO at inducible promoters correlates with increased transcript levels during gene induction [70]. A recent study demonstrated that loss of Tup1 sumoylation during amino acid starvation results in increased transcription of starvation-responsive genes, stabilization of Tup1’s presence at promoter regions, and enhanced removal of transcription complexes [71]. Here, we found that most of the genes with altered expression in the sumoylation-deficient Tup1^K270R^ mutant showed increased expression during hyperosmotic stress.

We find it interesting that the sumoylation-deficient Cyc8^4KtoR^ mutant exhibited increased expression for an even larger, yet distinct set of genes. This raises a few questions. Is there any mechanistic cross talk in the transcriptional effects exerted by sumoylation of Tup1 and Cyc8? Alternatively, are the gene expression effects in the Cyc8^4KtoR^ mutant due solely to inclusion formation of the Tup1-Cyc8 complex? We think it is possible that sumoylation of Cyc8 during hyperosmotic stress serves multiple purposes. Transient sumoylation of Cyc8 may act in a canonical manner to alter protein-protein interactions with other transcriptional regulators or machinery. Our data support an additional role for SUMO as a protective factor that prevents persistent inclusion formation of both Tup1 and Cyc8, which would allow for appropriate transcriptional control during hyperosmotic stress.

### The possibility that other glutamine-rich proteins are sumoylated during hyperosmotic stress

From the MS analysis in Fig 2, we primarily observed increased sumoylation for Cyc8 and its partner Tup1 during hyperosmotic stress. There are two plausible explanations for why this could be. First, Cyc8 has one of the longest polyglutamine tracts among yeast proteins. Upstream of Cyc8’s long polyglutamine tract is an extended glutamine-alanine repeat tract of 62 residues. Only Cyc8 has the unique combination of a long glutamine-alanine repeat sequence immediately followed by an extensive polyglutamine stretch. This makes it distinct among yeast glutamine-rich proteins. Second, we employed a label-free MS approach to identify proteins with increased sumoylation during hyperosmotic stress. This method may have not detected sumoylation changes in low abundance glutamine-rich proteins. Additional quantitative comparative studies will be needed to reveal the extent of glutamine-rich proteins that are sumoylated, especially those proteins that are lower in abundance than Cyc8.

It was recently reported in a MS study that other transcription-associated proteins in addition to Cyc8 and Tup1 are sumoylated during hyperosmotic stress [13]. Other than Cyc8-Tup1, we did not identify any of the additional reported proteins in our MS analysis (S1 Table). Although our study and the Lewicki *et al* study appear similar in terms of strains and experimental approach (purification of His_6_-tagged sumoylated proteins and analysis of abundance changes by peptide (spectral) counts), there are differences in how the experiments were performed. First, we used a yeast strain with the endogenous yeast *SMT3* gene modified with the *His*_*6*_*-FLAG* sequence, which allowed normal expression of the tagged SUMO from the endogenous *SMT3* promoter as the only source of SUMO. By contrast, Lewicki *et al* used a yeast strain with the endogenous *SMT3* gene intact but with additional overexpression of the *His*_*6*_*-FLAG-SMT3* from the exceptionally strong *GAL1* promoter on a high-copy plasmid. It is possible that overexpression of *His*_*6*_*-FLAG-SMT3* increased the pool of free SUMO available for sumoylation of other proteins, thus uncovering additional sumoylated proteins. Second, the cell lysis conditions were different between the two studies. The highly denaturing conditions we used (8M urea, 50mM Tris pH 8.0, 0.05% SDS) were selected because we found they robustly disrupt protein-protein and protein-DNA complexes. The conditions used by Lewicki *et al* might not have been as robust and it is possible that some complexes remained intact during cell lysis and nonsumoylated proteins copurified with sumoylated proteins. Lastly, it is possible that protein amounts and peptide separation prior to analysis by MS led to us identifying fewer proteins in our analysis. We add that, when we applied our statistical analysis to the data from Lewicki *et al*, Cyc8 and Tup1 were also the major proteins identified by their analysis (our analysis available upon request).

### The dynamics and nature of endogenous expression versus overexpression of Cyc8

Prior to our work here, the main pieces of evidence supporting Cyc8 as an inclusion-prone protein were studies that overexpressed C-terminal fragments of Cyc8 containing its glutamine-rich PrD. Overexpression of Cyc8’s PrD fragment induced the [OCT+] prion [35] and led to the formation of detergent-insoluble amyloid inclusions [53]. Notably, the portions of Cyc8 used in those studies lacked the Tup1-interaction region, which we show is necessary for Cyc8 sumoylation (Fig 3C). We find it interesting that overexpression of these sumoylation-deficient forms of Cyc8 leads to the formation of Cyc8’s prion state and SDS-resistant amyloid inclusions.

While we think overexpression studies can be informative about protein misfolding, overexpression of truncated proteins can also force inclusion states that might not normally occur under endogenous expression of full-length proteins. Prior to our studies, it was not clear whether normally expressed full-length Cyc8 had the capacity to form inclusions. Thus, an important finding of our studies is that we discovered an endogenous *in vivo* transition state of Cyc8 that forms inclusions upon hyperosmotic stress if Cyc8 is not sumoylated. The highly correlated dynamics of Cyc8 sumoylation support a model whereby sumoylation prevents persistent Cyc8 inclusion formation during hyperosmotic stress.

One question that emerges from our studies is why are endogenously expressed full-length Cyc8^4KtoR^ inclusions SDS-sensitive and reversible whereas overexpressed truncated Cyc8 PrD inclusions are amyloidogenic and persistent? We think it is important to consider that inclusions formed from proteins via self-association of glutamine-rich regions, or other intrinsically disordered domains, do not always progress into amyloid states [66,67]. An emerging model is that amyloid inclusions represent a solid-phase state that is relatively static in nature, whereas non-amyloid inclusions represent a liquid-phase state that is highly dynamic [66,67]. By this model, inclusions that adopt a liquid-phase state can mature into a solid-phase amyloid state only when conditions are right (e.g. when the concentration of the proteins is sufficiently high or when one of the proteins acquires a mutation increasing its aggregation propensity).

In the case of Cyc8, there could be numerous factors that influence the balance between a liquid-phase and solid-phase state. As discussed above, the level of expression (endogenous expression versus overexpression) and the use of truncated versus full-length forms are important factors that can explain the differences between our study and previous studies [35,53]. In addition, we observed that strains expressing a dominant negative allele of Hsp70 exhibit more persistent sumoylation-deficient Cyc8^4KtoR^ inclusions, suggesting that the Hsp70 protein folding machinery can contribute to the dynamic nature of these inclusions. Importantly, another key factor that likely influences the nature of endogenous Cyc8^4KtoR^ inclusions is the ability of the cell to adapt to hyperosmotic stress by activating accumulation of intracellular osmolytes and chemical chaperones via the Hog1 pathway. To induce amyloid formation, it is necessary to highly overexpress the Cyc8 PrD for >24hours [53]. By contrast, endogenously expressed Cyc8^4KtoR^ forms inclusions only during the ~40 minutes of hyperosmotic stress that occur before the Hog1 pathway allows the cell to fully adapt to the stress. Even in the absence of Hog1 function, cells still adapt to hyperosmotic stress [58], and so it is not surprising that Cyc8^4KtoR^ inclusions do resolve in *hog1*Δ cells (Fig 10C). Overall, the conditions during hyperosmotic stress under which endogenously expressed Cyc8^4KtoR^ forms inclusions are transient and likely not of sufficient duration to allow maturation to amyloids. We suspect that, if the proper genetic conditions could be found to completely ablate the cell’s ability to adapt to hyperosmotic stress, endogenous Cyc8^4KtoR^ inclusions might progress into an amyloid state. Future studies will be necessary to explore this important physiological aspect of Cyc8 inclusion biology.

### Broader implications of sumoylation as a protective solubilizing factor

The high solubility of SUMO and its ability as a fusion protein to promote the solubility of recombinant proteins has led to important biotechnological uses [47,48]. How SUMO is used to promote protein solubility physiologically is an emerging field of study [43,44,45,46]. In fact, a very recent study that parallels ours found that sumoylation altered the prionogenic inclusion formation of CPEB3, an important translation factor involved in synaptic plasticity, allowing it to switch from a translational activator to repressor [72]. Furthermore, many neurodegenerative disorders are causally linked to protein aggregation [73], and SUMO dysregulation is associated with many these disorders [74]. We think that understanding the role of sumoylation as a protective solubilizing factor has important implications, not only for fundamental cellular protein homeostasis, but also for neurodegenerative disease progression.

## Materials and Methods

### Yeast strains and plasmids

Yeast strains and plasmids used in this study are listed in S3 Table. Standard yeast genetic methods were used for these studies [75]. *His*_*6*_*-FLAG-SMT3* strains were generated by integrating a *His*_*6*_*-FLAG-SMT3*::*HIS3MX6* PCR fragment at the endogenous *SMT3* locus. Replacement of endogenous *SMT3* was confirmed by colony PCR and western analyses for both tagged and untagged Smt3. All gene deletions were verified by colony PCR.

### Growth and stress conditions

Cells were grown to a density of ~1.5x10^7^ cells/ml at 30°C in yeast complete (YC) media prior to stress induction. All 0 time point samples were collected before stress induction. For hyperosmotic stress, equal volumes of culture and YC+2.4M sorbitol were combined for a final concentration of 1.2M sorbitol. For heat stress, culture flasks were submerged in a 42°C water bath to rapidly warm the culture and were then moved to shaking platform 42°C incubator. For ethanol stress, equal volumes of culture and YC+20% ethanol were combined for a final concentration of 10% v/v ethanol. Induction of Cyc8^441-677^ and Rnq1 was performed by growing cells in YC with 3% raffinose to mid-log phase at 30°C to a density of ~2x10^6^ cells/ml, then inducing expression with 3% galactose at 30°C for 24 hours.

### Sumoylated protein purification

15ml aliquots of cells were collected at each time point after stress and flash frozen in liquid nitrogen. Harvested cells were lysed by vortexing with glass beads at 4°C in lysis buffer (8M urea, 50mM Tris pH 8.0, 0.05% SDS with 2mM PMSF and 10mM NEM). An aliquot representing 5% of the input was set aside. Cell lysates were incubated with TALON resin (Novagen) overnight at 4°C. The resin was washed 3x with wash buffer (8M urea, 50mM Tris pH 8.0, 200mM NaCl, 0.05% SDS, 5mM imidazole). Sumoylated proteins were eluted from the column by addition of loading buffer (8M urea, 10mM MOPS, 10mM EDTA, 1% SDS, 0.01% bromophenol blue, pH 6.8) and incubation at 65°C for 10 minutes.

### Western analyses

Sumoylated proteins were resolved by SDS-PAGE using 4–20% gradient gels. Western analyses were performed with rabbit anti-Smt3 (1:5000, a gift from Xiaolan Zhao), mouse anti-FLAG (1:2500, Sigma), mouse anti-HSV (1:2500, Novagen), mouse anti-HA (1:2500, Sigma), mouse anti-Pgk1 (1:5000, Abcam), or rabbit anti-GFP (1:2500, Sigma).

### Mass spectrometry analyses

Sumoylated proteins from cells exposed to 0 or 15 minutes of hyperosmotic stress were enriched by metal affinity chromatography as described above. Samples were run 1 cm into a 4–20% SDS-PAGE gel and slices were excised. Proteins in the gel slices were digested with trypsin and the digestion products were desalted and dried by vacuum centrifugation. Dried peptide mixtures were resuspended in 7μL of 0.1% formic acid. 5μL was analyzed using an LTQ OrbiTrap mass spectrometer (Thermo Scientific). Complete MS methods were performed as previously described [24].

The protein database search algorithm X!Tandem [76] was used to identify peptides from the Saccharomyces Genome Database (http://www.yeastgenome.org). Peptide false discovery rates were measured using Peptide Prophet [77]. Identified peptides were filtered using Peptide Prophet scores of ≥0.55 (~5% error rate). The entire dataset is in S1 Table (whole data (PP≥ 0.55) tab). The significance of the changes in peptide counts between 0 and 15 minutes of hyperosmotic stress was determined by a two-tailed, homoscedastic student’s t-test. Final data was filtered by a *p* value ≤0.01 and a ≥3-fold change in summed peptide counts, and is shown in S1 Table (≥ 3 fold changes (Fig 2B)).

### Microarray analyses

Duplicate biological cultures were grown from isolates of independent transformations. Duplicate cultures (a and b) were grown at 30°C in YC to a density of ~1.75x10^7^ cells/ml and 0, 30, and 60 minute hyperosmotic stress samples were harvested by fast filtration and flash frozen in liquid nitrogen. Total RNA was prepared from the cells by hot acid phenol extraction as described on the Dunham lab website (http://dunham.gs.washington.edu/protocols.shtml). Total RNA was labeled using the Quick Amp RNA labeling kit (Agilent) and Cy3- and Cy5-CTP (GE Healthcare). Labeled RNA was purified with the RNeasy mini kit (Qiagen). Yield and dye incorporation were measured with a Nanodrop spectrophotometer (Thermo Scientific). Cy3- and Cy5-labeled samples were combined to obtain ≥1pmol dye and cRNA in each channel. Labeled cRNA combinations were mixed with blocking agent and fragmentation buffer (Agilent), then incubated at 60°C for 30 minutes in the dark. Hi-RPM hybridization buffer (Agilent) was used to stop the reaction. Samples were hybridized to yeast gene expression 8x15K microarray slides (Agilent) at 65°C overnight. Gasket slides were removed in 6X SSPE/0.005% N-lauroylsarcosine. Array slides were washed 1 minute in 6X SSPE/0.005% N-lauroylsarcosine, 1 minute in 0.6X SSPE/0.005% N-lauroylsarcosine, and 30 seconds in acetonitrile. Data were acquired using an Agilent scanner and feature-extracted and normalized with the Agilent software using default settings. All raw microarray data are available from the Princeton Microarray Database (http://puma.princeton.edu) and the Gene Expression Omnibus (http://www.ncbi.nlm.nih.gov/geo) with accession number GSE57476. These data are included in S2 Table.

Average linkage hierarchical clustering was performed with Cluster 3.0 ([78], http://rana.lbl.gov/EisenSoftware.htm), using the similarity metric “Correlation (uncentered)”, and the cluster data were visualized with Java TreeView (http://jtreeview.sourceforge.net/). We observed that one strain (Tup1^K270R^/Cyc8, biological replicate a) contained a small 18.0 Kb region of aneuploidy from YDR211W-YDR261C, based on increased expression levels across all time points and for all genes within the region. We removed all genes within this region from the analysis. To evaluate experimental variation in sample handling and microarray procedures, we analyzed the distribution of the ratios at each feature of a “wt x wt” array in which wild-type, unstressed replicate samples were cohybridized. The distribution of log_2_-transformed gene expression ratios was centered at 0 with a standard deviation of 0.22. We used this data to define a threshold for significant gene expression changes of 3 standard deviations from the mean, which corresponded to ±0.65 log_2_ units, or a 1.57-fold change in gene expression. Mutant effect ratios were determined by subtracting the values of wild-type time points from the corresponding mutant time points (in log_2_ space).

### Fluorescence microscopy

Aliquots of cells at each time point after hyperosmotic stress were removed, fixed in 4% paraformaldehyde solution for 15 minutes at room temperature and then washed with PBS. Cells were imaged on a Nikon Eclipse 90i with a 100X objective, filters for GFP (HC HiSN 0 Shift filter set with excitation wavelength (450–490 nm), dichroic mirror (495 nm), and emission filter (500–550 nm)), and a Photometrics Cool Snap HQ2 cooled CCD camera with NIS-Elements acquisition software.

### Confocal microscopy

Confocal 3D images were acquired using a dual point-scanning Nikon A1R-si microscope equipped with a PInano Piezo stage (MCL), using a 60x PlanApo VC oil objective NA 1.40. Calculations of the fluorescence Intensity and Image processing were performed using NIS-Elements software.

### Structured illumination microscopy (SIM)

For SIM imaging, cells were grown as described above and seeded on Concanavalin A (Sigma) coated plates, then imaged live in real time with and without hyperosmotic stress. Images were acquired using a Nikon nSIM microscope in 3D mode with a 488nm and 561nm lasers. A 100x oil TIRF objective (NA 1.49) was used for the imaging. Prior to imaging the point-spread function was visualized with 100 nm fluorescence beads in order to adjust the correction ring of the objective to the coverslip thickness. For each super resolution image raw data were examined for the optical grid pattern presence. The final image was reconstructed using NIS-Elements software (Nikon).

### Semi-denaturing detergent agarose gel electrophoresis

Cells were grown in YC to a density of ~1.5x10^7^ cells/ml at 30°C. A volume of 15ml was collected for Cyc8-GFP strains and 5ml was collected for galactose-induced strains. All cells were flash frozen on dry ice and ethanol. Cells were lysed by vortexing with glass beads at 4°C in a nondenaturing buffer (100mM Tris pH 7.5, 200mM NaCl, 1mM EDTA, 5% glycerol, 1mM DTT, 1% NP-40, 250U/ml Benzonase nuclease, 1mM MgCl_2_, 2mM PMSF, and 10mM NEM). Lysates were centrifuged for 2 minutes at 3,000 rpm to pellet unlysed cells, and the supernatant was mixed with 4X SDD-AGE loading buffer (2X TAE, 20% glycerol, 8% SDS, bromophenol blue) and incubated at room temperature for 5 minutes before loading. Samples were run on a 1.5% agarose gel containing 0.1% SDS for 6 hours at 4°C and transferred to nitrocellulose by capillary transfer as previously described [61]. Western blotting was performed with anti-GFP to visualize GFP- and YFP-tagged proteins.

### Image processing

All blots were scanned using an Epson Perfection V350 Photo scanner at 300 dpi. All images were processed with a Mac iMac or Pro computer (Apple) using Photoshop CS or CS4 (Adobe).

## Supporting Information

S1 Fig Gene expression of the double sumoylation-deficient mutant is similar to that of the Cyc8 sumoylation-deficient mutant; Related to Fig 5. (**A**) Flocculation of *tup1*Δ, *TUP1-3HA*, *TUP1*^*K270R*^*-3HA*, *cyc8*Δ, *CYC8-3HSV*, and *CYC*^*4KtoR*^*-3HSV* cells. Cultures were grown in rich media overnight to saturation. Cells were imaged by microscopy. (**B**) Plots depicting the correlation of double sumoylation-deficient mutant gene expression (Tup1^K270R^Cyc8^4KtoR^) and single sumoylation-deficient mutant gene expression (Tup1^K270R^ or Cyc8^4KtoR^). Gene expression data are from the unstressed time points of the data shown in Fig 5A and S2 Table. (**C**) Changes in gene expression for 16 example genes often used in the literature at the 0 minute time point (unstressed condition). Data represent log_2_-transformed gene expression ratios averaged across replicate comparisons. (**D**) Changes in gene expression for 16 example genes often used in the literature after 30 and 60 minutes after 1.2M sorbitol addition. Data represent mutant-effect ratios from gene expression changes. (**E**) Hierarchical clustering of mutant-effect ratios from gene expression changes that are ≥2-fold in Tup1^K270R^ cells during 0, 30, and 60 minutes of hyperosmotic stress. (**F**) Hierarchical clustering of mutant-effect ratios from gene expression changes that are ≥2-fold in Cyc8^4KtoR^ cells during 0, 30, and 60 minutes of hyperosmotic stress. (**G**) Quantitative RT-PCT data for *HXT6* and *GSY1* over 0, 30, and 60 minutes of hyperosmotic stress (1.2M sorbitol). Values are the average of 3 separate isolates. Mutant-effect ratios (m.e.r.) are listed for comparison. (**H**) Full gene expression data for cluster in Fig 5B. Data represent log_2_-transformed gene expression ratios averaged across replicate comparisons.
(TIF)

S2 Fig Cyc8 and Tup1 sequences. (**A**) Domain schematic of Cyc8 with the polyglutamine tract and glutamine-rich PrD (residues 441–677) shown in green, and the sumoylation sites identified in this study shown in blue. TPR indicates tetratricopeptide repeats. Sequence of Cyc8. Disorder prediction of Cyc8 using IUPred [79]. (**B**) Domain schematic of Tup1 with the glutamine-rich regions shown in green, and the sumoylation site shown in blue. WD40 repeats are noted. Sequence of Tup1. Disorder prediction of Tup1 using IUPred [79].
(TIF)

S1 Table Full data for mass spectrometry experiments in Fig 2A. (XLS)

S2 Table Full data for transcript microarray analyses in Fig 4. (XLS)

S3 Table Yeast strains and plasmids used in this study. (DOC)

S1 Text Methods for quantitative RT-PCR in S1G Fig. (DOC)
