# Supplementary figures and images for: Dynamic Sumoylation of a Conserved Transcription Corepressor Prevents Persistent Inclusion Formation during Hyperosmotic Stress
Source: PLoS Genet. 2016 Jan 22;12(1):e1005809. doi: 10.1371/journal.pgen.1005809 (PMC4723248; doi:10.1371/journal.pgen.1005809)

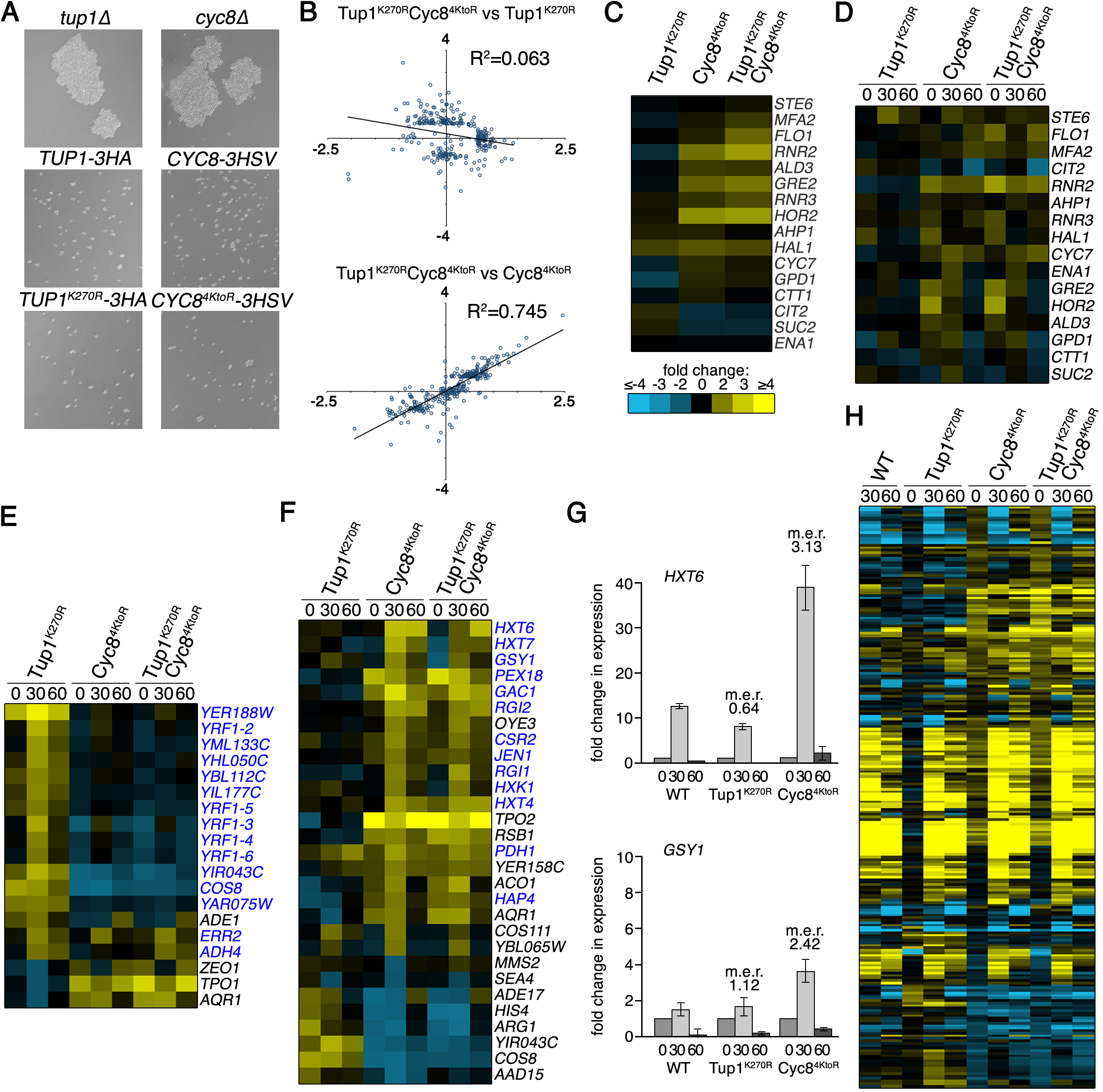

Supplement: S1 Fig — (A) Flocculation of tup1Δ, TUP1-3HA, TUP1K270R-3HA, cyc8Δ, CYC8-3HSV, and CYC4KtoR-3HSV cells. Cultures were grown in rich media overnight to saturation. Cells were imaged by microscopy. (B) Plots depicting the correlation of double sumoylation-deficient mutant gene expression (Tup1K270RCyc84KtoR) and single sumoylation-deficient mutant gene expression (Tup1K270R or Cyc84KtoR). Gene expression data are from the unstressed time points of the data shown in Fig 5A and S2 Table. (C) Changes in gene expression for 16 example genes often used in the literature at the 0 minute time point (unstressed condition). Data represent log2-transformed gene expression ratios averaged across replicate comparisons. (D) Changes in gene expression for 16 example genes often used in the literature after 30 and 60 minutes after 1.2M sorbitol addition. Data represent mutant-effect ratios from gene expression changes. (E) Hierarchical clustering of mutant-effect ratios from gene expression changes that are ≥2-fold in Tup1K270R cells during 0, 30, and 60 minutes of hyperosmotic stress. (F) Hierarchical clustering of mutant-effect ratios from gene expression changes that are ≥2-fold in Cyc84KtoR cells during 0, 30, and 60 minutes of hyperosmotic stress. (G) Quantitative RT-PCT data for HXT6 and GSY1 over 0, 30, and 60 minutes of hyperosmotic stress (1.2M sorbitol). Values are the average of 3 separate isolates. Mutant-effect ratios (m.e.r.) are listed for comparison. (H) Full gene expression data for cluster in Fig 5B. Data represent log2-transformed gene expression ratios averaged across replicate comparisons. (TIF) [file pgen.1005809.s001.tif]

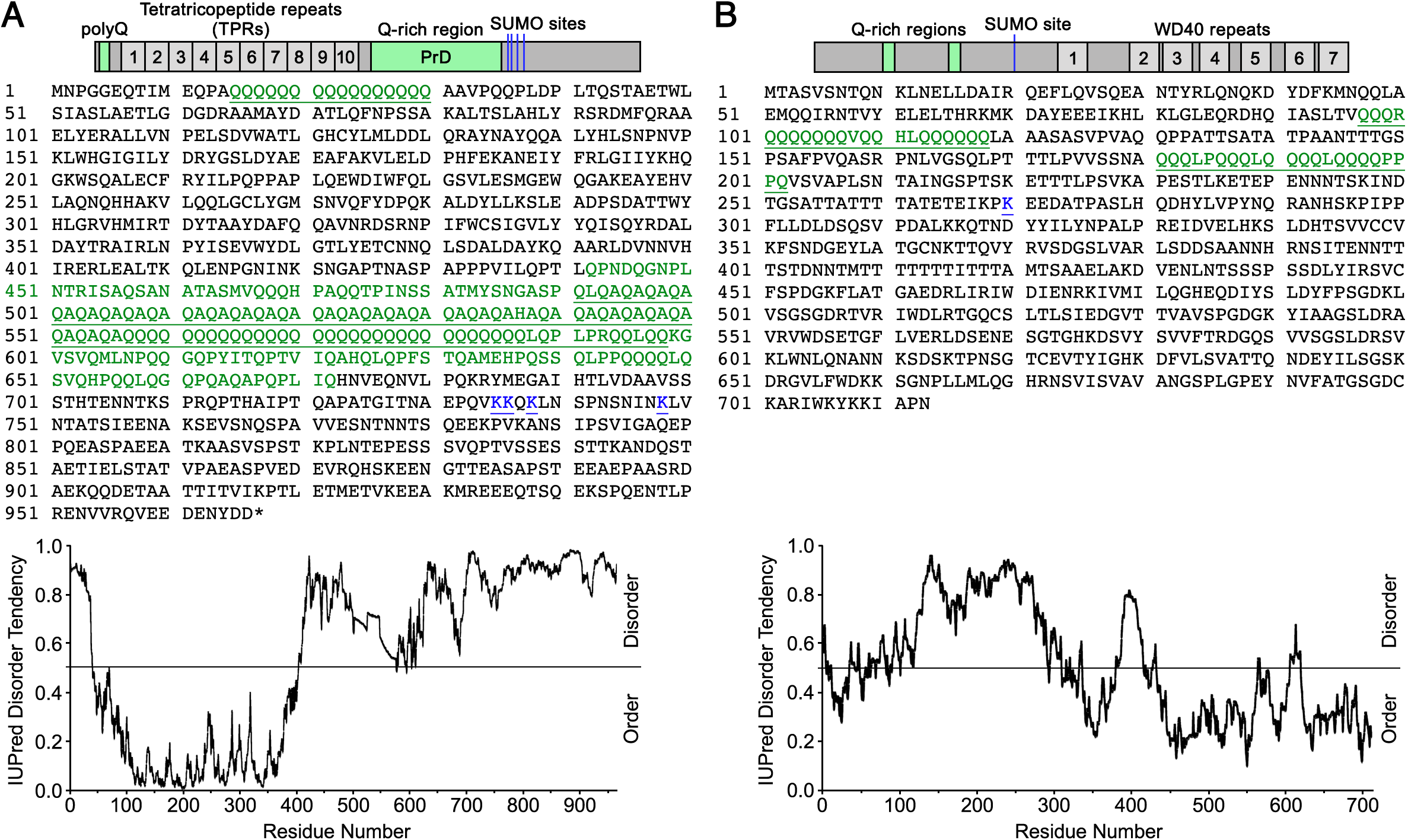

Supplement: S2 Fig — (A) Domain schematic of Cyc8 with the polyglutamine tract and glutamine-rich PrD (residues 441–677) shown in green, and the sumoylation sites identified in this study shown in blue. TPR indicates tetratricopeptide repeats. Sequence of Cyc8. Disorder prediction of Cyc8 using IUPred [79]. (B) Domain schematic of Tup1 with the glutamine-rich regions shown in green, and the sumoylation site shown in blue. WD40 repeats are noted. Sequence of Tup1. Disorder prediction of Tup1 using IUPred [79]. (TIF) [file pgen.1005809.s002.tif]
