# Supplementary material for: Dynamic Sumoylation of a Conserved Transcription Corepressor Prevents Persistent Inclusion Formation during Hyperosmotic Stress
Source: PLoS Genet. 2016 Jan 22;12(1):e1005809. doi: 10.1371/journal.pgen.1005809 (PMC4723248; doi:10.1371/journal.pgen.1005809)
Supplement: S3 Table — (DOC) [file pgen.1005809.s005.doc]

**Table S3: Yeast strains and plasmids used in this study**

Yeast strains

| **Strain** | **Genotype** | **Reference** |
| --- | --- | --- |
| BY4741 | *met15∆0, his3∆1, ura3∆0, leu2∆0* | [3] |
| RGY4136 | *met15∆0, his3∆1, ura3∆0, leu2∆0, P_GAL1_-RNQ1 URA3 2µ* | [4] |
| RGY5266 | *met15∆0, his3∆1, ura3∆0, leu2∆0, 6His-FLAG-SMT3::HIS3MX6* | This study |
| RGY5643 | *met15∆0, his3∆1, ura3∆0, leu2∆0, 6His-FLAG-SMT3::HIS3MX6, TUP1-3HA::NatMX* | This study |
| RGY5642 | *met15∆0, his3∆1, ura3∆0, leu2∆0, TUP1-3HA::NatMX* | This study |
| RGY5750 | *met15∆0, his3∆1, ura3∆0, leu2∆0, 6His-FLAG-SMT3::HIS3MX6, CYC8-3HSV::URA3* | This study |
| RGY5722 | *met15∆0, his3∆1, ura3∆0, leu2∆0, CYC8-3HSV::URA3* | This study |
| RGY5641 | *met15∆0, his3∆1, ura3∆0, leu2∆0, 6His-FLAG-SMT3::HIS3MX6, tup1∆* | This study |
| RGY5645 | *met15∆0, his3∆1, ura3∆0, leu2∆0, 6His-FLAG-SMT3::HIS3MX6, cyc8∆* | This study |
| RGY5702 | *met15∆0, his3∆1, ura3∆0, leu2∆0, 6His-FLAG-SMT3::HIS3MX6, tup1∆, cyc8∆* | This study |
| RGY5758 | *met15∆0, his3∆1, ura3∆0, leu2∆0, 6His-FLAG-SMT3::HIS3MX6, tup1∆::TUP1-3HA::URA3, cyc8∆::CYC8::LEU2* | This study |
| RGY5759 | *met15∆0, his3∆1, ura3∆0, leu2∆0, 6His-FLAG-SMT3::HIS3MX6, tup1∆::TUP1(K270R)-3HA::URA3, cyc8∆::CYC8::LEU2* | This study |
| RGY5754 | *met15∆0, his3∆1, ura3∆0, leu2∆0, 6His-FLAG-SMT3::HIS3MX6, tup1∆::TUP1::URA3, cyc8∆::CYC8-3HSV::LEU2* | This study |
| RGY5756 | *met15∆0, his3∆1, ura3∆0, leu2∆0, 6His-FLAG-SMT3::HIS3MX6, tup1∆::TUP1::URA3, cyc8∆::CYC8(K735R,K736R,K738R,K748R)-3HSV::LEU2* | This study |
| RGY5704 | *met15∆0, his3∆1, ura3∆0, leu2∆0, 6His-FLAG-SMT3::HIS3MX6, siz1∆* | This study |
| RGY5744 | *met15∆0, his3∆1, ura3∆0, leu2∆0, 6His-FLAG-SMT3::HIS3MX6, siz1∆, CYC8-3HSV::URA3* | This study |
| RGY5746 | *met15∆0, his3∆1, ura3∆0, leu2∆0, 6His-FLAG-SMT3::HIS3MX6, siz1∆, TUP1-3HA::NatMX* | This study |
| RGY5705 | *met15∆0, his3∆1, ura3∆0, leu2∆0, 6His-FLAG-SMT3::HIS3MX6, siz2∆* | This study |
| RGY5707 | *met15∆0, his3∆1, ura3∆0, leu2∆0, 6His-FLAG-SMT3::HIS3MX6, cst9∆* | This study |
| RGY5749 | *met15∆0, his3∆1, ura3∆0, leu2∆0, 6His-FLAG-SMT3::HIS3MX6, MMS21-3HSV-IAA1.T10::KanMX, CYC1::AFB2::LEU2* | This study |
| RGY5711 | *met15∆0, his3∆1, ura3∆0, leu2∆0, 6His-FLAG-SMT3::HIS3MX6, ULP1-3HSV-IAA1.10::KanMX, CYC1::AFB2::LEU2* | This study |
| RGY5745 | *met15∆0, his3∆1, ura3∆0, leu2∆0, 6His-FLAG-SMT3::HIS3MX6, ULP1-3HSV-IAA1.10::KanMX, CYC1::AFB2::LEU2, CYC8-3HSV::URA3* | This study |
| RGY5747 | *met15∆0, his3∆1, ura3∆0, leu2∆0, 6His-FLAG-SMT3::HIS3MX6, ULP1-3HSV-IAA1.10::KanMX, CYC1::AFB2::LEU2, TUP1-3HA::NatMX* | This study |
| RGY5712 | *met15∆0, his3∆1, ura3∆0, leu2∆0, 6His-FLAG-SMT3::HIS3MX6, ULP2-3HSV-IAA1.10::KanMX, CYC1::AFB2::LEU2* | This study |
| RGY5753 | *met15∆0, his3∆1, ura3∆0, leu2∆0, 6His-FLAG-SMT3::HIS3MX6, TUP1::TUP1(73-713)-3HA::URA3* | This study |
| RGY5751 | *met15∆0, his3∆1, ura3∆0, leu2∆0, 6His-FLAG-SMT3::HIS3MX6, CYC8::CYC8(176-966)-3HSV::LEU2* | This study |
| RGY5762 | *met15∆0, his3∆1, ura3∆0, leu2∆0, 6His-FLAG-SMT3::HIS3MX6, tup1∆::TUP1::URA3, cyc8∆::CYC8::LEU2, isolate 1* | This study |
| RGY5763 | *met15∆0, his3∆1, ura3∆0, leu2∆0, 6His-FLAG-SMT3::HIS3MX6, tup1∆::TUP1::URA3, cyc8∆::CYC8::LEU2, isolate 2* | This study |
| RGY5765 | *met15∆0, his3∆1, ura3∆0, leu2∆0, 6His-FLAG-SMT3::HIS3MX6, tup1∆::TUP1(K270R)::URA3, cyc8∆::CYC8::LEU2, isolate 1* | This study |
| RGY5766 | *met15∆0, his3∆1, ura3∆0, leu2∆0, 6His-FLAG-SMT3::HIS3MX6, tup1∆::TUP1(K270R)::URA3, cyc8∆::CYC8::LEU2, isolate 2* | This study |
| RGY5768 | *met15∆0, his3∆1, ura3∆0, leu2∆0, 6His-FLAG-SMT3::HIS3MX6, tup1∆::TUP1::URA3, cyc8∆::CYC8(K735R,K736R,K738R,K748R)::LEU2, isolate 1* | This study |
| RGY5769 | *met15∆0, his3∆1, ura3∆0, leu2∆0, 6His-FLAG-SMT3::HIS3MX6, tup1∆::TUP1::URA3, cyc8∆::CYC8(K735R,K736R,K738R,K748R)::LEU2, isolate 2* | This study |
| RGY5771 | *met15∆0, his3∆1, ura3∆0, leu2∆0, 6His-FLAG-SMT3::HIS3MX6, tup1∆::TUP1(K270R)::URA3, cyc8∆::CYC8(K735R,K736R,K738R,K748R)::LEU2, isolate 1* | This study |
| RGY5772 | *met15∆0, his3∆1, ura3∆0, leu2∆0, 6His-FLAG-SMT3::HIS3MX6, tup1∆::TUP1(K270R)::URA3, cyc8∆::CYC8(K735R,K736R,K738R,K748R)::LEU2, isolate 2* | This study |
| RGY5812 | *met15∆0, his3∆1, ura3∆0, leu2∆0, 6His-FLAG-SMT3::HIS3MX6, tup1∆::TUP1::URA3, cyc8∆::CYC8-GFP::LEU2* | This study |
| RGY5813 | *met15∆0, his3∆1, ura3∆0, leu2∆0, 6His-FLAG-SMT3::HIS3MX6, tup1∆::TUP1(K270R)::URA3, cyc8∆::CYC8-GFP::LEU2* | This study |
| RGY5814 | *met15∆0, his3∆1, ura3∆0, leu2∆0, 6His-FLAG-SMT3::HIS3MX6, tup1∆::TUP1::URA3, cyc8∆::CYC8(K735R,K736R,K738R,K748R)-GFP::LEU2* | This study |
| RGY5815 | *met15∆0, his3∆1, ura3∆0, leu2∆0, 6His-FLAG-SMT3::HIS3MX6, tup1∆::TUP1(K270R)::URA3, cyc8∆::CYC8(K735R,K736R,K738R,K748R)-GFP::LEU2* | This study |
| RGY5816 | *met15∆0, his3∆1, ura3∆0, leu2∆0, 6His-FLAG-SMT3::HIS3MX6, tup1∆::TUP1-GFP::URA3, cyc8∆::CYC8::LEU2* | This study |
| RGY5817 | *met15∆0, his3∆1, ura3∆0, leu2∆0, 6His-FLAG-SMT3::HIS3MX6, tup1∆::TUP1(K270R)-GFP::URA3, cyc8∆::CYC8::LEU2* | This study |
| RGY5818 | *met15∆0, his3∆1, ura3∆0, leu2∆0, 6His-FLAG-SMT3::HIS3MX6, tup1∆::TUP1-GFP::URA3, cyc8∆::CYC8(K735R,K736R,K738R,K748R)::LEU2* | This study |
| RGY5819 | *met15∆0, his3∆1, ura3∆0, leu2∆0, 6His-FLAG-SMT3::HIS3MX6, tup1∆::TUP1(K270R)-GFP::URA3, cyc8∆::CYC8(K735R,K736R,K738R,K748R)::LEU2* | This study |
| RGY5820 | *met15∆0, his3∆1, ura3∆0, leu2∆0, 6His-FLAG-SMT3::HIS3MX6, cyc8∆::CYC8-GFP::LEU2* | This study |
| RGY5821 | *met15∆0, his3∆1, ura3∆0, leu2∆0, 6His-FLAG-SMT3::HIS3MX6, cyc8∆::CYC8(∆441-677)-GFP::LEU2* | This study |
| RGY5822 | *met15∆0, his3∆1, ura3∆0, leu2∆0, 6His-FLAG-SMT3::HIS3MX6, cyc8∆::CYC8(K735R,K736R,K738R,K748R)-GFP::LEU2* | This study |
| RGY5823 | *met15∆0, his3∆1, ura3∆0, leu2∆0, 6His-FLAG-SMT3::HIS3MX6, cyc8∆::CYC8(∆441-677,K735R,K736R,K738R,K748R)-GFP::LEU2* | This study |
| RGY5820 | *met15∆0, his3∆1, ura3∆0, leu2∆0, 6His-FLAG-SMT3::HIS3MX6, cyc8∆::CYC8-GFP::LEU2, hog1∆* | This study |
| RGY5822 | *met15∆0, his3∆1, ura3∆0, leu2∆0, 6His-FLAG-SMT3::HIS3MX6, cyc8∆::CYC8(K735R,K736R,K738R,K748R)-GFP::LEU2, hog1∆* | This study |

Plasmids

| **Name** | **Encoded protein** | **Parent vector** | **Reference** |
| --- | --- | --- | --- |
| pRG3990 | *P_GPD_-AFB2* | pGP5G-ccdB | [5] |
| pRG1673 | *P_GAL1_-RNQ1-YFP* | pRS416 | [6] |
| pRG4190 | *pAG424GAL-CYC8 PrD-EYFP* | pAG424GAL-ccdB-EYFP | [7] |
| pRG4043 | *TUP1* | pRS406 | This study |
| pRG4129 | *TUP1(K270R)* | pRS406 | This study |
| pRG4059 | *TUP1-3HA* | pRS406 | This study |
| pRG4060 | *TUP1(K270R)-3HA* | pRS406 | This study |
| pRG4111 | *TUP1(73-713)-3HA* | pRS406 | This study |
| pRG4086 | *TUP1-GFP* | pRS406 | This study |
| pRG4087 | *TUP1(K270R)-GFP* | pRS406 | This study |
| pRG4020 | *CYC8* | pRS405 | This study |
| pRG4130 | *CYC8(K735R,K736R,K738R,K748R)* | pRS405 | This study |
| pRG4052 | *CYC8-3HA* | pRS405 | This study |
| pRG4079 | *CYC8(1-900)-3HA* | pRS405 | This study |
| pRG4080 | *CYC8(1-830)-3HA* | pRS405 | This study |
| pRG4081 | *CYC8(1-745)-3HA* | pRS405 | This study |
| pRG4082 | *CYC8(1-715)-3HA* | pRS405 | This study |
| pRG4084 | *CYC8-3HSV* | pRS405 | This study |
| pRG4113 | *CYC8(K735R,K736R,K738R,K748R)-3HSV* | pRS405 | This study |
| pRG4110 | *CYC8(176-966)-3HSV* | pRS405 | This study |
| pRG4085 | *CYC8-GFP* | pRS405 | This study |
| pRG4128 | *CYC8(K735R,K736R,K738R,K748R)-GFP* | pRS405 | This study |
| pRG4193 | *CYC8(∆441-677,K735R,K736R,K738R,K748R)-GFP* | pRS405 | This study |
| pRG4240 | *CYC8-mCherry* | pRS416 | This study |
| pRG4241 | *CYC8(K735R,K736R,K738R,K748R)-mCherry* | pRS416 | This study |
| pRG4242 | *TUP1-mCherry* | pRS406 | This study |
| pRG4244 | *CYC8(K735R,K736R,K738R,K748R)-GFP-SV40NLS* | pRS405 | This study |

**REFERENCES**

1. Livak KJ, Schmittgen TD (2001) Analysis of relative gene expression data using real-time quantitative PCR and the 2(-Delta Delta C(T)) Method. Methods 25: 402-408.

2. Dosztanyi Z, Csizmok V, Tompa P, Simon I (2005) IUPred: web server for the prediction of intrinsically unstructured regions of proteins based on estimated energy content. Bioinformatics 21: 3433-3434.

3. Brachmann CB, Davies A, Cost GJ, Caputo E, Li J, et al. (1998) Designer deletion strains derived from Saccharomyces cerevisiae S288C: a useful set of strains and plasmids for PCR-mediated gene disruption and other applications. Yeast 14: 115-132.

4. Konopka CA, Locke MN, Gallagher PS, Pham N, Hart MP, et al. (2011) A yeast model for polyalanine-expansion aggregation and toxicity. Mol Biol Cell 22: 1971-1984.

5. Havens KA, Guseman JM, Jang SS, Pierre-Jerome E, Bolten N, et al. (2012) A synthetic approach reveals extensive tunability of auxin signaling. Plant Physiol 160: 135-142.

6. Douglas PM, Treusch S, Ren HY, Halfmann R, Duennwald ML, et al. (2008) Chaperone-dependent amyloid assembly protects cells from prion toxicity. Proc Natl Acad Sci U S A 105: 7206-7211.

7. Alberti S, Halfmann R, King O, Kapila A, Lindquist S (2009) A systematic survey identifies prions and illuminates sequence features of prionogenic proteins. Cell 137: 146-158.
