# Supplementary material for: Dynamic Sumoylation of a Conserved Transcription Corepressor Prevents Persistent Inclusion Formation during Hyperosmotic Stress
Source: PLoS Genet. 2016 Jan 22;12(1):e1005809. doi: 10.1371/journal.pgen.1005809 (PMC4723248; doi:10.1371/journal.pgen.1005809)
Supplement: S1 Text — (DOC) [file pgen.1005809.s006.doc]

**Supplemental information for:**

**Dynamic sumoylation of a conserved transcription corepressor prevents persistent inclusion formation during hyperosmotic stress**

Michelle L. Oeser^1,2^, Triana Amen^3,4^, Cory M. Nadel^2^, Amanda I. Bradley^1^, Benjamin J. Reed^2^, Ramon D. Jones^2^, Janani Gopalan^2^, Daniel Kaganovich^4^, and Richard G. Gardner^1,2^*

^1^Molecular and Cellular Biology Program

^2^Department of Pharmacology

University of Washington, Seattle, WA, 98195, USA

^3^Alexander Grass Center for Bioengineering

^4^Department of Cell and Developmental Biology

Alexander Silberman Institute of Life Sciences

Hebrew University of Jerusalem

Jerusalem, Israel 91904

*Corresponding author

E-mail: [gardnerr@uw.edu](mailto:gardnerr@uw.edu)

**SUPPLEMENTAL METHODS**

**Quantitative Reverse Transcription (qRT) PCR**

Triplicate biological cultures of wild-type, Tup1^K270R^, and Cyc8^4KtoR^ cells were grown at 30˚C in YC to a density of ~1.75x10^7^ cells/ml. Samples were harvested at 0, 30, and 60 minutes after hyperosmotic stress by fast filtration and flash frozen in liquid nitrogen. Total RNA was prepared from the cells by hot acid phenol extraction as described on the Dunham lab website (<http://dunham.gs.washington.edu/protocols.shtml>). The quality of RNA was checked by examining the A_260_/A_280_ ratio with a Nanodrop 2000c Spectrophotometer (Thermo Scientific). Equivalent amounts of total RNA were reverse-transcribed using Superscript III reverse transcriptase (Invitrogen) according to the manufacturer instructions using Random Hexamers.

For each sample of cDNA, we queried three different genes: *HXT6*, a high-affinity glucose transporter, *GSY1*, a glycogen synthase and *ACT1* as a control. For the actin control *ACT1* Forward: 5’ TGGCCGGTAGAGATTTGACTGACT 3’; Reverse: 5’ TCGAAGTCCAAGGCGACGTAACAT 3’. *HXT6* Forward: 5’ GTCCTGCATCCATGACTGCTTGT 3’; Reverse: 5’ GGAATTGGAGCCCATGTAGTAGC 3’. *GSY1* Forward: 5’ TCCTTGGTTTGGATTATGACGAGT 3’; Reverse: 5’ ACCGAACCCAGAAACGTTTGTTG 3’.

Real-time PCR was performed on a Realplex 2 qPCR machine (Eppendorf). The PCR mixture (10.2µl total) contained 5µL of KAPA SYBR FAST qPCR Master Mix (KAPA Biosystems), 1µl of cDNA, 0.1µl of 10µM forward primer, 0.1µl of 10µM reverse primer, and 4µl RNAse-DNAse free water. The protocol for PCR consisted of one denaturation at 95°C for 3 minutes followed by 40 cycles of denaturation at 95°C for 15 seconds, annealing at 55°C for 15 seconds, and extension at 72°C for 15 seconds. Data was collected at the end of each step repeat. For each sample, *ACT1* was used as an endogenous control to normalize mRNA levels. All CT data was normalized to *ACT1* using the 2^-∆∆CT^ method [1].

∆CT_control_ = [Target Gene Delete_ACT1_- WT­_ACT1_]

∆CT_Experiment_ = [Target Gene Delete_Experiment_ - WT_Experiement_]

Difference = ∆CT_Control_-∆CT_Experiment_

Normalized ∆∆CT values = 2^(∆CTControl-∆CTExperiment)^

CT values from ∆CT_Control_ are reactions in which *ACT1* is being observed in each cDNA sample. CT values from ∆CT_Experiment_ are reactions in which the target genes, *HXT6* or *GSY1,* are being observed in each cDNA sample. The normalized values were plotted on a bar graph for each gene of interest at the 0, 30, and 60 minutes after hyperosmotic stress time points. Each 0 time point for wild-type, Tup1^K270R^, Cyc8^4KtoR^ cultures was arbitrarily set to a value of 1. Fold changes from the 0 time point were determined for the 30 and 60 minute time points. Error bars are the standard deviation between triplicate samples at the 30 and 60 minute time points.

**REFERENCES**

1. Livak KJ, Schmittgen TD (2001) Analysis of relative gene expression data using real-time quantitative PCR and the 2(-Delta Delta C(T)) Method. Methods 25: 402-408.

2. Dosztanyi Z, Csizmok V, Tompa P, Simon I (2005) IUPred: web server for the prediction of intrinsically unstructured regions of proteins based on estimated energy content. Bioinformatics 21: 3433-3434.

3. Brachmann CB, Davies A, Cost GJ, Caputo E, Li J, et al. (1998) Designer deletion strains derived from Saccharomyces cerevisiae S288C: a useful set of strains and plasmids for PCR-mediated gene disruption and other applications. Yeast 14: 115-132.

4. Konopka CA, Locke MN, Gallagher PS, Pham N, Hart MP, et al. (2011) A yeast model for polyalanine-expansion aggregation and toxicity. Mol Biol Cell 22: 1971-1984.

5. Havens KA, Guseman JM, Jang SS, Pierre-Jerome E, Bolten N, et al. (2012) A synthetic approach reveals extensive tunability of auxin signaling. Plant Physiol 160: 135-142.

6. Douglas PM, Treusch S, Ren HY, Halfmann R, Duennwald ML, et al. (2008) Chaperone-dependent amyloid assembly protects cells from prion toxicity. Proc Natl Acad Sci U S A 105: 7206-7211.

7. Alberti S, Halfmann R, King O, Kapila A, Lindquist S (2009) A systematic survey identifies prions and illuminates sequence features of prionogenic proteins. Cell 137: 146-158.
